# Efficacy and safety of acupuncture in post-stroke constipation: a systematic review and meta-analysis

**DOI:** 10.3389/fnins.2023.1275452

**Published:** 2023-09-26

**Authors:** Tianye Sun, Kaiyue Wang, Lili Li, Mingyuan Yan, Lin Zou, Mi Zhang, Songyi Yang, Jing Wu, Jinmin Liu

**Affiliations:** ^1^Beijing University of Chinese Medicine, Beijing, China; ^2^Dongzhimen Hospital, Beijing University of Chinese Medicine, Beijing, China; ^3^Dongfang Hospital, Beijing University of Chinese Medicine, Beijing, China

**Keywords:** acupuncture, post-stroke constipation, efficacy, safety, meta-analysis, systematic review, randomized clinical trials

## Abstract

**Background and objective:**

Post-stroke constipation (PSC) is a common complication of strokes that seriously affects the recovery and quality of life of patients, and effective treatments are needed. Acupuncture is a viable treatment option, but current evidence is insufficient to support its efficacy and safety. This study aims to evaluate the efficacy and safety of acupuncture in the treatment of PSC.

**Methods:**

A systematic search of eight databases was conducted to identify PSC-related randomized clinical trials from the inception of each database through May 2023. Methodological quality assessment was conducted by RoB 2.0, meta-analysis was conducted by RevMan 5.3 and Stata 15.1, and evidence quality was evaluated by GRADE. Moreover, reporting quality of acupuncture interventions was assessed using the Standards for Reporting Interventions in Clinical Trials of Acupuncture (STRICTA).

**Results:**

Thirty RCTs involving 2,220 patients were identified. We found that acupuncture was superior to conventional treatment (CT) in improving total responder rate [risk ratio (*RR*): 1.16, 95% confidence interval (CI): 1.09 to 1.25, *p* < 0.0001], decreasing constipation symptom scores [standardized mean difference (*SMD*): -0.65, 95% CI: −0.83 to −0.46, *p* < 0.00001], increasing serum P substance (SP) levels (*SMD*: 1.92, 95% CI: 0.47 to 3.36, *p* = 0.009), reducing the time to first bowel movement (BM) (*SMD*: -1.19, 95% CI: −2.13 to −0.25, *p* = 0.01), and lowing serum vasoactive intestinal peptide (VIP) levels (*SMD*: –2.11, 95% CI: −3.83 to −0.38, *p* = 0.02). Furthermore, acupuncture plus CT was superior regarding total responder rate (*RR*: 1.26, 95% CI: 1.17 to 1.35, *p* < 0.00001), serum SP levels (*SMD*: 2.00, 95% CI: 1.65–2.35, *p* < 0.00001), time to first BM (*SMD*: –2.08, 95% CI: −2.44 to −1.71, *p* < 0.00001), and serum VIP levels (*SMD*: –1.71, 95% CI: −2.24 to −1.18, *p* < 0.00001). However, regarding Bristol Stool Scale (BSS) score, acupuncture plus CT was superior to CT (*SMD*: -2.48, 95% CI: −3.22 to −1.73, *p* < 0.00001), while there was no statistically significant difference between acupuncture and CT (*SMD*: 0.28, 95% CI: −0.02 to 0.58, *p* = 0.07). Acupuncture causes fewer AEs than CT (*RR*: 0.13, 95% CI: 0.06 to 0.26, *p* < 0.00001), though there was no statistically significant difference between acupuncture plus CT vs. CT (*RR*: 1.30, 95% CI: 0.60 to 2.84, *p* = 0.51).

**Conclusion:**

Acupuncture may be an effective and safe therapy for PSC. However, given the inferior quality of clinical data, additional well-designed RCTs are required to confirm these findings.

## 1. Introduction

Stroke is one of the top three global disease burdens (GBD 2019 Stroke Collaborators, 2021), and post-stroke constipation (PSC) is a common complication with a prevalence of 50–70% in stroke patients (Su et al., 2009; Liu Q. et al., 2018; Liu Z. et al., 2018). PSC seriously affects the treatment and rehabilitation of stroke (Harris and Chang, 2022), leading to decreased quality of life, prolonged hospitalization, and increased healthcare costs. PSC also induces or aggravates other complications like post-stroke depression and may lead to recurrent stroke and death (Sumida et al., 2019), placing a substantial burden on the national healthcare system.

The exact mechanism of PSC is unclear. Studies suggest a close relationship with autonomic dysfunction (Liu Q. et al., 2018; Liu Z. et al., 2018), multidrug usage (Su et al., 2009), dietary changes, and reduced activity (Lim et al., 2015). Recently, the brain-gut axis has received much attention as a bi-directional channel between the gastrointestinal tract and the autonomic nerves of the central nervous system that uses a variety of neurotransmitters, brain-gut peptides, and gut microbes (Carabotti et al., 2015; Camilleri, 2021; Xu et al., 2021), which are considered relevant to the development of PSC. Because of the lack of clinical and basic research related to PSC, current treatment strategies are mostly based on clinical practice guidelines for functional constipation (Chang et al., 2022, 2023), such as the use of laxatives, 5-hydroxytryptamine type 4 agonists, and enemas. However, relief is usually temporary, and side effects such as bloating, diarrhea, colon damage, melanosis coli, and cardiovascular adverse events cannot be ignored (Gilsenan et al., 2019; Chang et al., 2022, 2023). Safer and more effective alternative treatments are urgently needed.

Acupuncture is a complementary and alternative medicine of Chinese origin, of which the most common forms include manual acupuncture (MA), electroacupuncture (EA), moxibustion, and warm acupuncture (WA). Studies demonstrate that acupuncture is a relatively safe alternative to laxatives that effectively alleviates gastrointestinal and neurological symptoms in patients (Wang et al., 2015; Liu et al., 2016; Pei et al., 2020; Lu et al., 2022; Zheng et al., 2022). Moreover, electrophysiology studies have shown that acupuncture regulates autonomic nerves and gastrointestinal hormones by transmitting signals from somatic stimulation to the central nervous system *via* the upper spinal cord, thereby affecting gastrointestinal tract function (Takahashi, 2013).

Clearly, acupuncture may be an effective and safe complementary and alternative therapy to improve PSC. Previous meta-analyses (Yang et al., 2014; Tang et al., 2020) have investigated the therapeutic effects of acupuncture on PSC, but their conclusions require further validation because of deficiencies in outcome indicators, included literature, and controls for confounding factors. Therefore, to further confirm the efficacy and safety of the treatment, we conducted a comprehensive evaluation of the available clinical evidence on the latest randomized clinical trial (RCT) data of acupuncture for PSC.

## 2. Materials and methods

### 2.1. Registration

The protocol for this systematic review and meta-analysis was registered in PROSPERO (No. CRD42023425087; https://www.crd.york.ac.uk/PROSPERO/), and followed the Preferred Reporting Items for Systematic Reviews and Meta-Analyses (PRISMA) guidelines (Page et al., 2021).

### 2.2. Literature search

Two researchers (STY and WKY) independently searched PubMed, EMBASE, Cochrane Library, Web of Science, Chinese National Knowledge Infrastructure (CNKI), SinoMed, Chinese Science and Technique Journals Database (VIP), Wanfang Database, and two clinical trial registries (ClinicalTrials.gov and the Chinese Clinical Trial Registry) from study inception to May 9, 2023. The language restriction was English and Chinese. The search terms were “Constipation” “Dyschezia” “Colonic Inertia” “Astriction” “Stroke” “Cerebral Infarction” “Cerebral Hemorrhage” “Cerebrovascular Disorders” “Post stroke constipation” “Acupuncture” “Electroacupuncture” “Dry Needling” “Moxibustion” “Integrated Chinese and western medicine” “Complementary Therapies” and related terms. We also consulted citations from relevant systematic reviews. Details of the search strategies were shown in Supplementary File S1.

### 2.3. Eligibility criteria

#### 2.3.1. Study types

Prospective parallel RCTs of acupuncture for the treatment of PSC.

#### 2.3.2. Participants

Participants diagnosed with PSC, without gender or age restrictions. Stroke was diagnosed by Magnetic Resonance Imaging or Computed Tomography scan, and constipation was diagnosed by Rome II, Rome III, Rome IVcriteria, diagnostic and curative effect standard of Chinese medicine disease and syndrome, or the guiding principles for clinical research of new Chinese medicine (The State Administration of Traditional Chinese Medicine, 1994; Drossman, 1999, 2006, 2016; Zheng, 2002).

#### 2.3.3. Interventions

The experimental group received acupuncture as monotherapy or as an adjunct to conventional treatment (CT), including manual acupuncture (MA), electroacupuncture (EA), warm-acupuncture (WA), moxibustion, dry needling, auricular acupuncture, and laser acupuncture, etc.

#### 2.3.4. Comparisons

Participants in the control group were treated with CT or sham acupuncture. Conventional treatment was limited to fiber, osmotic laxatives (e.g., polyethylene glycol, lactulose, magnesium oxide), stimulant laxatives (e.g., senna, sodium picosulfate, bisacodyl), gastrointestinal prokinetic drugs (e.g., prucalopride, cisapride, mosapride), and secretagogues (e.g., lubiprostone, linaclotide, plecanatide). There were no restrictions on dosage, route of administration, or treatment duration.

#### 2.3.5. Outcomes

The included studies reported at least one primary outcome of total responder rate and constipation symptom score. The secondary outcomes included time to first bowel movement (BM), serum vasoactive intestinal peptide (VIP) levels, serum P substance (SP) levels, Bristol Stool Scale (BSS) score, and adverse events (AEs).

The total responder rate was defined as the proportion of patients whose symptoms improved, and we accepted the definitions reported in each study. The constipation symptom score referred to the Constipation Symptoms Scale designed by Anorectal Surgery Group of the Chinese Medical Association’s Surgery Branch (2005); the scale considers criteria of difficulty in defecation, duration of defecation, BSS score, incomplete defecation, frequency of defecation, and bloating.

### 2.4. Exclusion criteria

We excluded the following studies: (1) studies with other Chinese medicine treatments, such as Chinese herbs, massage, acupoint injection, auricular acupressure, scraping, cupping, and catgut embedding therapy, and trials that compared different acupuncture types; (2) studies with incomplete or incorrect data; (3) studies involving patients with disorders of consciousness, cognitive impairment, or serious diseases of the heart, liver, and kidney hematopoietic system; (4) studies without full-text availability.

### 2.5. Data extraction

Two researchers (WKY and LLL) independently screened the titles, abstracts, and full texts of the retrieved studies for eligibility and independently extracted the data of the final included literature. Disagreements were resolved by mutual negotiation or by consultation with a third researcher (STY). The following information was extracted: authors, publication year, general information, participants’ characteristics, details of interventions (type of acupuncture, acupoints, frequency, duration of treatment, retention time of acupuncture), and outcomes.

### 2.6. Risk of bias assessment

Two researchers (ZL and ZM) independently assessed the methodological quality of the included studies using the Cochrane Risk of Bias Tool 2.0 (RoB 2.0) (Sterne et al., 2019), which contains six aspects: randomization, deviations from the intended interventions, missing outcome data, measurement of the outcome, selective outcome reporting, and overall bias. Each aspect was evaluated as “low risk of bias,” “some concerns,” or “high risk of bias.” Disagreements were resolved by mutual negotiation or by consultation with a third researcher (YSY).

### 2.7. Quality of acupuncture treatment regimen in included trials

Two researchers (YMY and WJ) independently evaluated the reporting quality of interventions in each study with the Revised Standards for Reporting Interventions in Clinical Trials of Acupuncture (STRICTA) (MacPherson et al., 2010). The revised STRICTA consists of six items: acupuncture rationale, details of needling, treatment regimen, other components of treatment, practitioner background, and control intervention. Disagreements were resolved by mutual negotiation or by consultation with a third researcher (STY).

### 2.8. Statistical analysis

All statistical analyses were conducted using Review Manager 5.4 and Stata 15.1 software. Dichotomous outcomes were expressed as the risk ratio (*RR*) with 95% confidence interval (CI), while continuous outcomes were expressed as standardized mean difference (*SMD*) with 95% CI (Murad et al., 2019). Heterogeneity was assessed by the *χ*^2^ test and the *I^2^* statistic (Higgins et al., 2019). The fixed-effect model was used in cases with low heterogeneity (*p* > 0.1, *I^2^* < 50%), and the random-effect model was applied in cases with substantial heterogeneity (*p* ≤ 0.1, *I^2^* ≥ 50%) (Higgins et al., 2019; Murad et al., 2019).

Sensitivity analysis was conducted by excluding individual studies to investigate the stability of the results. Subgroup analysis was conducted to investigate the potential causes of heterogeneity with four prespecified aspects: (1) treatment duration (<2 weeks, ≥2 weeks); (2) acupuncture frequency (<1 time/day, ≥1 time/day); (3) needle retention time (<30 min, ≥30 min); (4) types of control interventions (osmotic laxatives, stimulant laxatives, gastrointestinal prokinetic drugs, secretagogues). In addition, a funnel plot and Egger’s test were applied to evaluate publication bias when the number of included studies was more than 10, and the trim-and-fill method was used to explore whether publication bias impacted the results. Descriptive analysis was performed if the data were not suitable for meta-analysis.

### 2.9. Quality of evidence

The GRADE (Grading of Recommendations Assessment, Development and Evaluatio) system (Liu, 2022) was used to rank the quality of evidence in five downgrading domains: risk of bias, inconsistency, indirectness, imprecision, and publication bias. The quality of evidence was classified into four grades: high, moderate, low, or very low.

## 3. Results

### 3.1. Identification of studies

**Figure 1:**
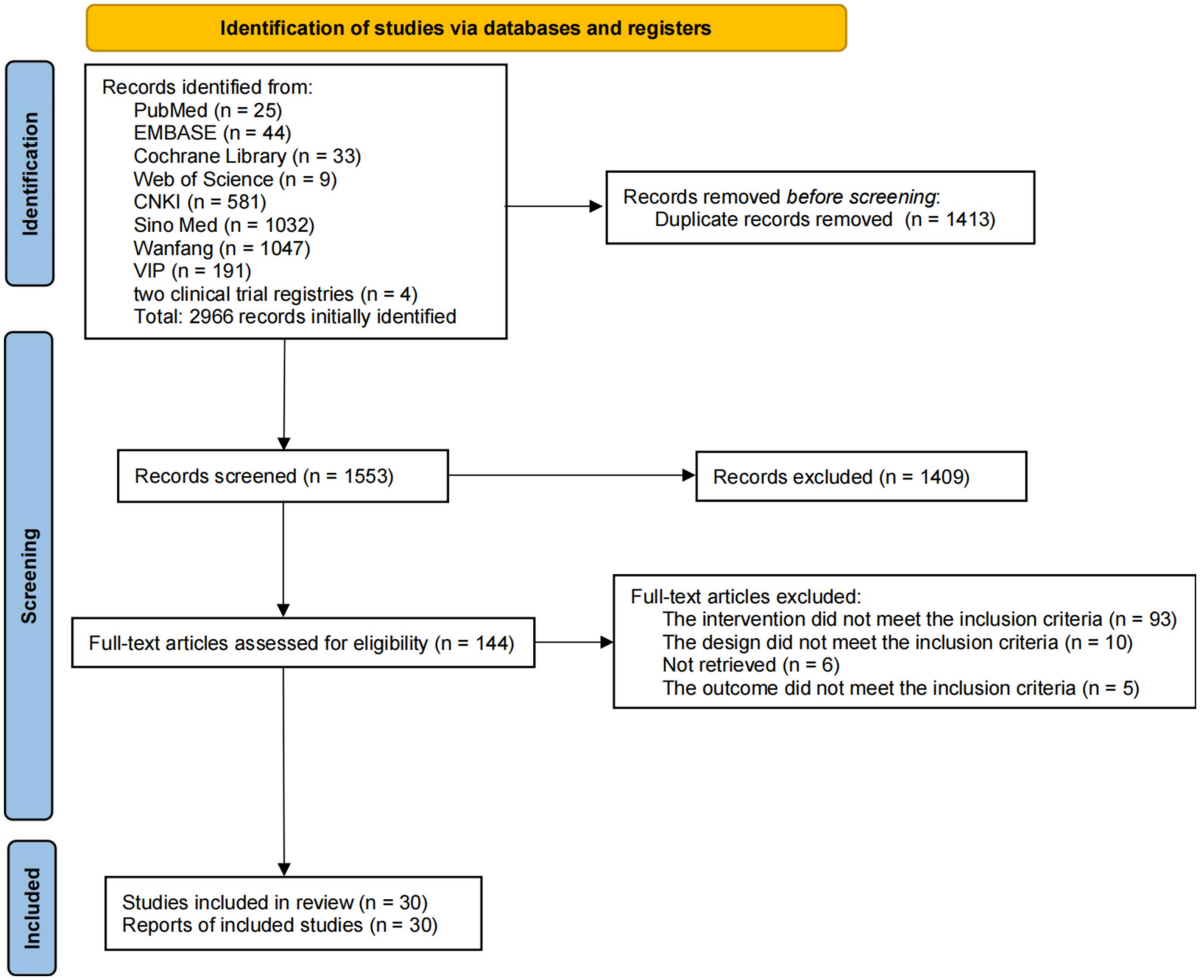
Literature selection process.

A total of 2,966 publications were retrieved from the eight databases and two clinical trial registries, and 1,413 duplicate publications were eliminated. After a review of the titles and abstracts, 1,409 publications were excluded, leaving 144 publications for secondary assessment. After reading the full text, 114 studies were eliminated (reasons for exclusion are shown in Supplementary Table S1), leaving 30 studies for inclusion (Zhou and Wang, 2001; Li and Song, 2005; Liu et al., 2008; Wang et al., 2008, 2019, 2021; Zhang et al., 2008, 2015; Cao and Sun, 2009; Shi, 2009; Zhu and Chen, 2010; Tian and Wang, 2012; Man, 2014; Yuan et al., 2014, 2021; Ma and Li, 2015; Song and Liu, 2015; Xie et al., 2016; Zhang, 2016; Gao et al., 2017; Li et al., 2018; Liu Q. et al., 2018; Liu Z. et al., 2018; Yang, 2018; Guan, 2019; Luo, 2019; Lu et al., 2020; Wu et al., 2020; Liu and Wang, 2022; Tian, 2022; Zhong et al., 2022; Figure 1).

### 3.2. Characteristics of the included studies

Thirty RCTs were included in the systematic review and meta-analysis. The 30 RCTs enrolled a total of 2,220 participants (*n* = 1,125 and *n* = 1,095 from the intervention and control groups, respectively), with sample sizes ranging from 40 to 110. All trials were single-center RCTs conducted in China and published in Chinese from 2001 to 2022. Twenty-seven studies (Zhou and Wang, 2001; Li and Song, 2005; Liu et al., 2008; Wang et al., 2008, 2019, 2021; Zhang et al., 2008, 2015; Cao and Sun, 2009; Shi, 2009; Tian and Wang, 2012; Man, 2014; Yuan et al., 2014; Ma and Li, 2015; Song and Liu, 2015; Xie et al., 2016; Zhang, 2016; Gao et al., 2017; Li et al., 2018; Liu Q. et al., 2018; Liu Z. et al., 2018; Yang, 2018; Guan, 2019; Luo, 2019; Wu et al., 2020; Liu and Wang, 2022; Tian, 2022; Zhong et al., 2022) were two-armed, while three studies (Zhu and Chen, 2010; Lu et al., 2020; Yuan et al., 2021) were three-armed. Of the three-arm studies, two (Zhu and Chen, 2010; Yuan et al., 2021) compared low stimulus vs. high stimulus vs. CT, and one (Lu et al., 2020) compared Tianshu (ST25) vs. Zhigou (SJ6) vs. CT.

Eleven studies compared MA with CT, six trials compared EA with CT, three trials compared WA with CT, one trial compared moxibustion vs. CT, seven trials compared MA plus CT vs. CT, and two trials compared moxibustion plus CT vs. CT. Control interventions included osmotic laxatives (polyethylene glycol, lactulose), stimulant laxatives (senna, bisacodyl, phenolphthalein), and gastrointestinal prokinetic drugs (cisapride). Table 1 displays the characteristics of the 30 studies.

**Table 1:** Characteristics of the included trials.

Study	Sample size (M/F); Mean age (years)		Interventions		Course of treatment	Outcomes
	**T**	**C**	**T**	**C**		
Zhong et al. (2022)	16/14; 59.73 ± 4.75	14/16; 60.40 ± 4.39	MA; FREQ = 5 sessions a week; NRT = 30 min	Lactulose oral solution, 30 ml before breakfast, qd	4 w	①⑦
Yuan et al. (2021)	14/16; 53.47 ± 5.20	16/14; 54.80 ± 6.94	MA; FREQ = 6 sessions a week; NRT = 30 min	Lactulose oral solution, 30 ml, qd, no medication on Sundays	2 w	①②⑥
Wang et al. (2021)	23/14; 52.78 ± 10.28	21/16; 53.03 ± 11.29	MA; FREQ = 1 session daily; NRT: BP-UE3 without needle retention, and 30 min for the rest of acupoints	Lactulose oral solution, 25 ml at breakfast, qd	10 d	①②③
Lu et al. (2020)	22/6; 63.75 ± 10.885 25/4; 64.69 ± 11.031	23/7; 61.83 ± 9.337	MA; FREQ = 1 session daily; NRT = 30 min	Lactulose oral solution, 10 ml in the morning, qd	2 w	①②④⑤⑥
Guan (2019)	NR	NR	MA; FREQ = 5 sessions a week; NRT = 30 min	Lactulose oral solution, in the morning, qd	4 w	②③
Wang et al. (2019)	27/22; 44 ± 5	31/18; 43 ± 5	EA; FREQ = 1 session daily; NRT = 30 min	Cisapride tablets, 5 mg, tid	4 w	①⑦
Luo (2019)	26/4; 54.5	25/5; 55.8	EA; FREQ = 5 sessions a week; NRT = 30 min	Folium Sennae, 3–5 g, qd, continuous or intermittent	3 w	①
Liu Q. et al. (2018)	22/28; 55.12 ± 8.54	27/23; 57.24 ± 10.91	MA; FREQ = 1 session daily; NRT = 30 min	Lactulose oral solution, 10 ml, tid	7 w	②⑦
Gao et al. (2017)	14/16; 56 ± 10	17/13; 57 ± 12	MA; FREQ = 1 session daily; NRT = 30 min	Lactulose oral solution, 20–30 ml in the morning, qd	6 w	②③④⑤⑦
Zhang (2016)	19/21; 60.3 ± 7.6	27/23; 58.8 ± 9.7	WA; FREQ = 1 session daily; NRT = 20 ~ 30 min	Phenolphthalein tablets, 100 mg, qd	2 w	①⑦
Song and Liu (2015)	24/16; 66.2 ± 3.3	27/13; 65.9 ± 4.7	WA; FREQ = 1 session daily; NRT: when the 1.5–2 cm moxa is burnt out	Phenolphthalein tablets, 100 mg, qd	3 w	①⑦
Ma and Li (2015)	20/15; 63.53 ± 12.78	19/12; 64.43 ± 14.32	moxibustion; FREQ = 5 sessions a week; NRT = 20 min	Lactulose oral solution, 10/15–30 ml, bid	4 w	①
Man (2014)	NR	NR	MA; FREQ = 6 sessions a week; NRT = 20 min	Phenolphthalein tablets, 200 mg, bid	2 w	①
Tian and Wang (2012)	16/14; 59.97 ± 9.5	17/13; 57.90 ± 9.2	EA; FREQ = 1 session daily; NRT = 30 min	Folium Sennae, 3–5 g, qd	2 w	①
Shi (2009)	23/17; 58 ± 7	22/18; 58 ± 6	MA; FREQ = 1 session daily; NRT = 30 min	Phenolphthalein tablets, 200 mg, qd	15 d	①
Cao and Sun (2009)	11/19; NR	8/12; NR	MA; FREQ = 1 session daily; NRT: NR	Bisacodyl enteric-coated tablets, 5 mg, qd	1 w	①
Liu et al. (2008)	19/16; 59.6 ± 8.23	21/14; 58.9 ± 8.18	EA; FREQ = 1 session daily; NRT = 30 min	Folium Sennae, 3–5 g, qd	10 d	①⑦
Zhang et al. (2008)	13/17; 62.6 ± 3.6	14/16; 62.1 ± 5.7	MA; FREQ = 1 session daily; NRT = 30 min	Folium Sennae, 3–5 g, qd	15 d	①
Wang et al. (2008)	22/18; 63.2 ± 3.74	23/17; 61.9 ± 4.65	EA; FREQ = 1 session daily; NRT = 30 min	Cisapride tablets, 10 mg, 30 min before a meal, bid	2 w	①
Li and Song (2005)	19/13; 65.2 ± 3.7	18/14; 64.9 ± 4.9	WA; FREQ = 1 session daily; NRT: when the 2.5–3 cm moxa is burnt out	Phenolphthalein tablets, 50 mg at bedtime, qd	15 d	①
Zhou and Wang (2001)	30	30	EA; FREQ = 5 sessions a week; NRT = 30 min	Folium Sennae, 3–5 g, qd	3 w	①⑦
Liu and Wang (2022)	16/20; 51.68 ± 7.43	18/18; 51.32 ± 8.14	MA + CT; FREQ = 1 session daily; NRT = 20 min	Lactulose oral solution, 30 ml, qd	20 d	①③⑦
Tian (2022)	19/11; 60.52 ± 6.34	18/12; 60.59 ± 6.31	MA + CT; FREQ = 1 session daily; NRT = 30 min	Phenolphthalein tablets, 50–200 mg, qd	1 w	①④⑦
Wu et al. (2020)	23/17; 55.30 ± 6.50	25/15; 55.00 ± 6.60	moxibustion + CT; FREQ = 1 session daily; NRT = 15–20 min	Lactulose oral solution, 10 ml, tid	14 d	①
Yang (2018)	23/32; 56.09 ± 10.64	25/30; 56.23 ± 10.70	MA + CT; FREQ = 1 session daily; NRT = 30 min	Lactulose oral solution, 20 ml, qd	1 m	①③④⑤⑥⑦
Li et al. (2018)	29/11; 62.64 ± 5.38	27/13; 62.71 ± 5.42	MA + CT; FREQ = 1 session daily; NRT = 30 min	Lactulose oral solution, 30 mL, qd	6 w	①④⑤⑥⑦
Xie et al. (2016)	16/14; 65.40 ± 9.89	17/13; 60.50 ± 10.47	MA + CT; FREQ = 1 session daily; NRT: NR	Lactulose oral solution, 15 ml, tid	2 w	①
Zhang et al. (2015)	21/19; 69.50 ± 7.208	22/18; 69.00 ± 8.901	MA + CT; FREQ = 1 session daily; NRT: NR	Macrogol 4,000 powder, 20 g, bid	1 w	①⑦
Yuan et al. (2014)	19/15; 67	20/18; 64	MA + CT; FREQ = 1 session daily; NRT = 120 min	Macrogol 4,000 powder, 10 g before breakfast and dinner, bid	2 w	①
Zhu and Chen (2010)	20/10; 57.96	22/8; 58.25	Moxibustion + CT; FREQ = 5 sessions a week; NRT > 120 min	Folium Sennae, 5 g, qd	3 w	①

bid, twice daily; C, control group; cm, centimeter; CT, conventional treatment; d, day; EA, electroacupuncture; F, female; FREQ, frequency; m, month; M, male; MA, manual acupuncture; NR, not reported; NRT, needle retention time; qd, once daily; T, treatment group; tid, thrice daily; w, week; WA, warm acupuncture; ① total responder rate; ② constipation symptom score; ③ time to first BM; ④ serum VIP levels; ⑤ serum SP levels; ⑥ BSS score; ⑦ adverse events.

### 3.3. Acupuncture protocol of the included studies

Eighteen studies (Zhang et al., 2008, 2015; Cao and Sun, 2009; Shi, 2009; Man, 2014; Yuan et al., 2014, 2021; Xie et al., 2016; Gao et al., 2017; Li et al., 2018; Liu Q. et al., 2018; Liu Z. et al., 2018; Yang, 2018; Guan, 2019; Lu et al., 2020; Wang et al., 2021; Liu and Wang, 2022; Tian, 2022; Zhong et al., 2022) used MA, six studies (Zhou and Wang, 2001; Liu et al., 2008; Wang et al., 2008, 2019; Tian and Wang, 2012; Luo, 2019) used EA, three studies (Li and Song, 2005; Song and Liu, 2015; Zhang, 2016) used WA, and three studies (Zhu and Chen, 2010; Ma and Li, 2015; Wu et al., 2020) used moxibustion. The frequency of treatment ranged from 5 to 7 sessions per week for a 1–7 week duration of treatment. Retention times ranged from 15 to 120 min. Twenty-five studies (Zhou and Wang, 2001; Li and Song, 2005; Liu et al., 2008; Wang et al., 2008, 2019; Zhang et al., 2008, 2015; Cao and Sun, 2009; Shi, 2009; Zhu and Chen, 2010; Tian and Wang, 2012; Yuan et al., 2014, 2021; Ma and Li, 2015; Xie et al., 2016; Zhang, 2016; Gao et al., 2017; Li et al., 2018; Liu Q. et al., 2018; Liu Z. et al., 2018; Yang, 2018; Luo, 2019; Lu et al., 2020; Wu et al., 2020; Liu and Wang, 2022; Zhong et al., 2022) applied fixed treatment protocols, three studies (Man, 2014; Song and Liu, 2015; Wang et al., 2021) applied individualized treatment protocols (fixed acupoints combined with acupoints based on symptoms and syndrome differentiation of Chinese medicine), and two (Guan, 2019; Tian, 2022) reported only representative acupoints. The most common acupoints were Tianshu (ST25), Zusanli (ST36), Qihai (RN6), Zhigou (SJ6), Zhongwan (RN12), Taichong (LR3), Shangjuxu (ST37), Neiguan (PC6), Danzhong (RN17), and Guanyuan (RN4). Frequency ranking of acupoints is shown in Supplementary Table S2.

### 3.4. STRICTA checklist for the included studies

The reporting quality of trial treatment protocols was evaluated by STRICTA with 17 items. As shown in Supplementary Figure S1, nearly all trials reported item 1a (acupuncture rationale), item 1b (reasoning for acupuncture treatment), item 3a (number of treatment sessions), item 3b (frequency and duration of treatment sessions), and item 6b (precise description of the control group). No studies mentioned item 4b (setting and context of treatment) and item 5 (acupuncturist’s background). The STRICTA checklist is provided in Supplementary Table S3.

### 3.5. Quality assessment

**Figure 2:**
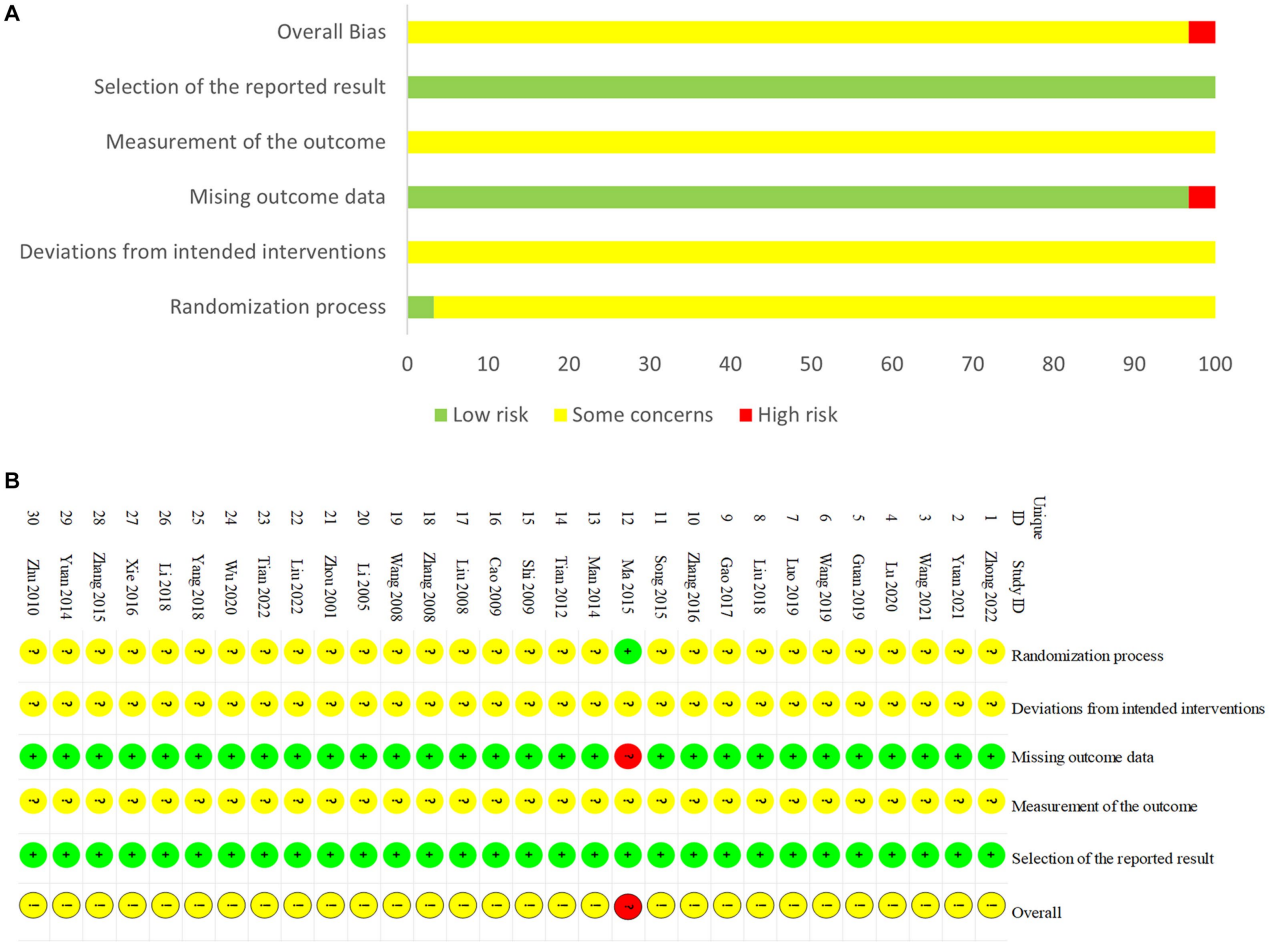
Risk of bias. **(A)** Risk of bias summary. **(B)** Risk of bias graph.

Regarding randomization, 16 studies (Wang et al., 2008, 2019, 2021; Zhang et al., 2008, 2015; Shi, 2009; Tian and Wang, 2012; Man, 2014; Yuan et al., 2014, 2021; Ma and Li, 2015; Gao et al., 2017; Luo, 2019; Lu et al., 2020; Tian, 2022; Zhong et al., 2022) provided a sufficient randomized sequence generation process. In addition, one study (Ma and Li, 2015) used consecutively numbered, opaque, sealed envelopes for allocation concealment and was evaluated as low risk. The remaining studies did not provide specific details of allocation concealment, and therefore we evaluated the risk of bias as unclear. Regarding deviations from the intended interventions, due to the specificity of acupuncture therapy, one study (Gao et al., 2017) implemented blinding of therapists, and the remaining 29 studies did not report the implementation of blinding. With respect to missing outcome data, one study (Ma and Li, 2015) reported missing visits but did not perform an intention-to-treat approach, which may affect the true outcome. The remaining 29 studies had no missing data, so we evaluated the risk of bias as low. Regarding outcome measurement, one study (Gao et al., 2017) implemented blinding of outcome assessors, and the remaining 29 studies did not mention the implementation of blinding. Regarding selective outcome reporting, the expected outcomes of all studies are fully reported. The risk of bias for all trials is shown in Figure 2.

### 3.6. Primary outcomes

#### 3.6.1. Total responder rate

##### 3.6.1.1. Acupuncture vs. CT

**Figure 3:**
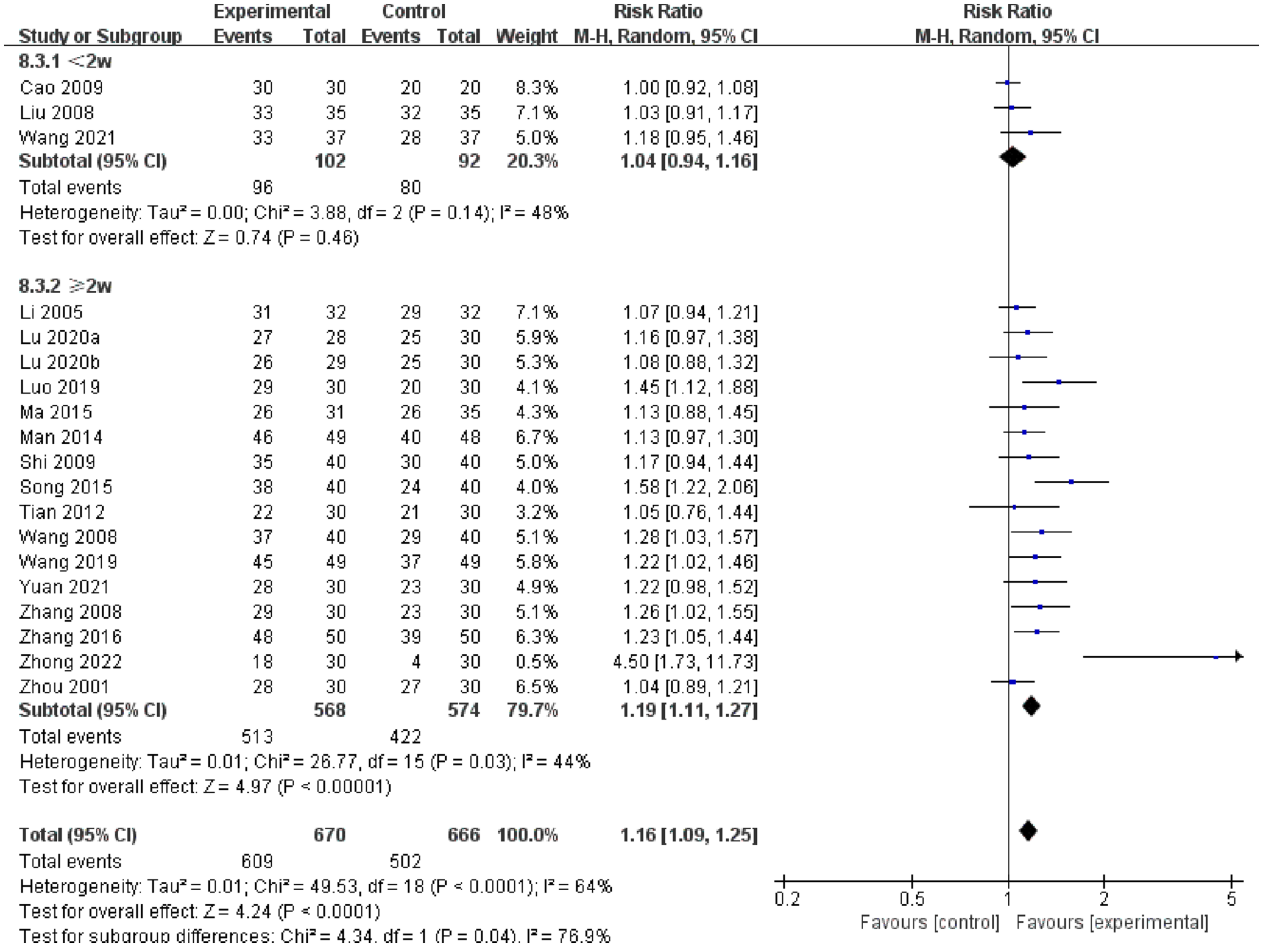
Forest plot of acupuncture on total responder rate.

Nineteen studies compared the total responder rates for acupuncture and CT, and the random-effects model was used for the meta-analysis because of the high heterogeneity among studies (*p* < 0.0001, *I*^2^ = 64%). We found that acupuncture was superior to CT in total responder rate (*RR* 1.16, 95% CI: 1.09–1.25, *p* < 0.0001), and sensitivity analysis revealed that results were robust against the exclusion of any one study (Supplementary Figure S2A). Furthermore, we conducted a subgroup meta-analysis to determine if treatment duration influences the efficacy of acupuncture treatment; we found that acupuncture treatment durations of <2 weeks did not offer a benefit, while treatment durations ≥2 weeks were more effective. Notably, the difference in interaction effect between these two subgroups was highly significant. These results indicate an optimal treatment duration of ≥2 weeks (Figure 3).

**Figure 4:**
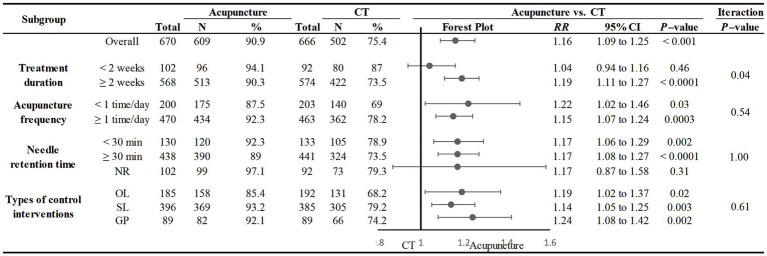
Subgroup analysis of total responder rate comparing acupuncture and CT.

We also conducted subgroup analyses of the effects of acupuncture frequency (< 1 time/day, ≥ 1 time/day), needle retention time (time < 30 min, time ≥ 30 min, not reported), and the types of control interventions (OL, SL, GP) on the efficacy of acupuncture. We found that most subgroups were consistent with the overall findings, suggesting that the acupuncture frequency and types of medication did not significantly influence the positive effect of acupuncture in treating PSC. However, within the subgroup analysis in which needle retention time was not mentioned, acupuncture was not superior to CT (*RR*: 1.17, 95% CI: 0.87–1.58, *p* = 0.31) (Figure 4).

##### 3.6.1.2. Acupuncture plus CT vs. CT

**Figure 5:**
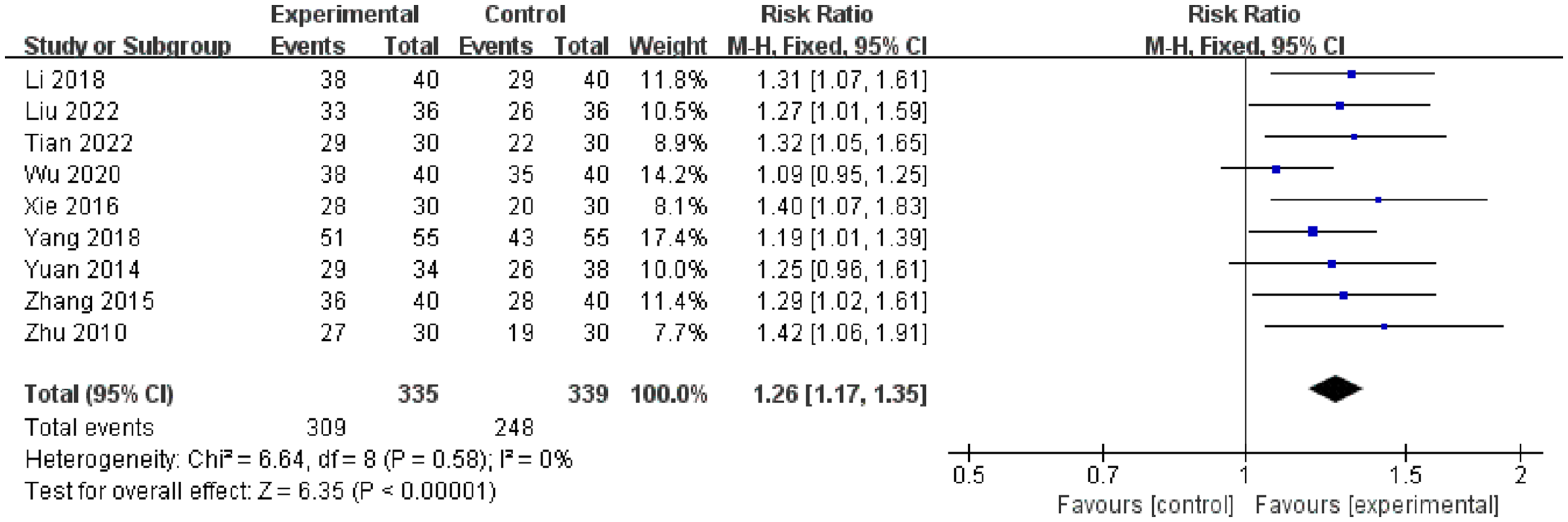
Forest plot of acupuncture plus CT on total responder rate.

Nine studies compared the total responder rate for acupuncture plus CT vs. CT, and the fixed-effects model was used because there was no heterogeneity among the studies (*p* = 0.58, *I*^2^ = 0%). We found that acupuncture plus CT was superior to CT regarding total responder rate (*RR*: 1.26, 95% CI: 1.17 to 1.35, *p* < 0.00001) (Figure 5), and sensitivity analysis revealed that results were robust against the exclusion of any one study (Supplementary Figure S2B).

**Figure 6:**
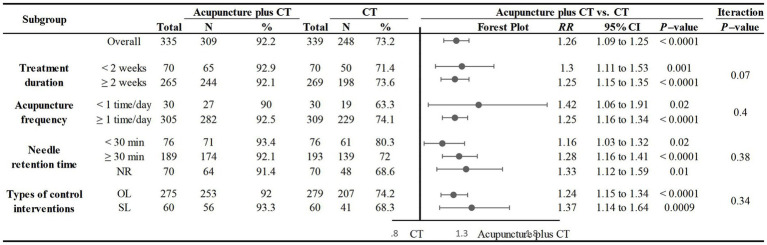
Subgroup analysis of total responder rate comparing acupuncture plus CT vs. CT.

In addition, we conducted subgroup analyses based on acupuncture frequency (<1 time/day, ≥1 time/day), treatment duration (<2 weeks, ≥2 weeks), needle retention time (time < 30 min, time ≥ 30 min, not reported), and the types of control interventions (OL, SL) to determine the influence of these characteristics on the efficacy of acupuncture. We found that all subgroups were consistent with the overall findings, suggesting that these characteristics did not significantly affect the positive effect of acupuncture in treating PSC (Figure 6).

#### 3.6.2. Constipation symptom score

##### 3.6.2.1. Acupuncture vs. CT

**Figure 7:**
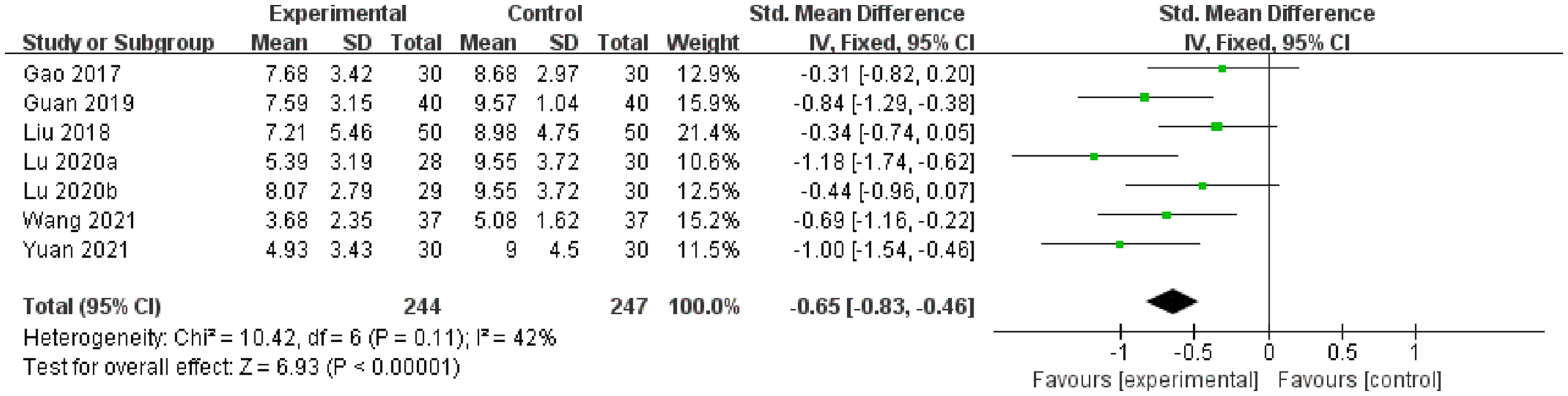
Forest plot of the constipation symptom score.

Seven studies compared constipation symptom scores for acupuncture vs. CT, and the fixed-effects model was used because of the low heterogeneity among the studies (*p* = 0.11, *I*^2^ = 42%). As shown in Figure 7, acupuncture reduced constipation symptom scores to a greater extent than did CT (*SMD*: -0.65, 95% CI: −0.83 to −0.46, *p* < 0.00001), and sensitivity analysis revealed that results were robust against the exclusion of any one study (Supplementary Figure S2C).

### 3.7. Secondary outcomes

#### 3.7.1. Time to first BM

##### 3.7.1.1. Acupuncture vs. CT

**Figure 8:**
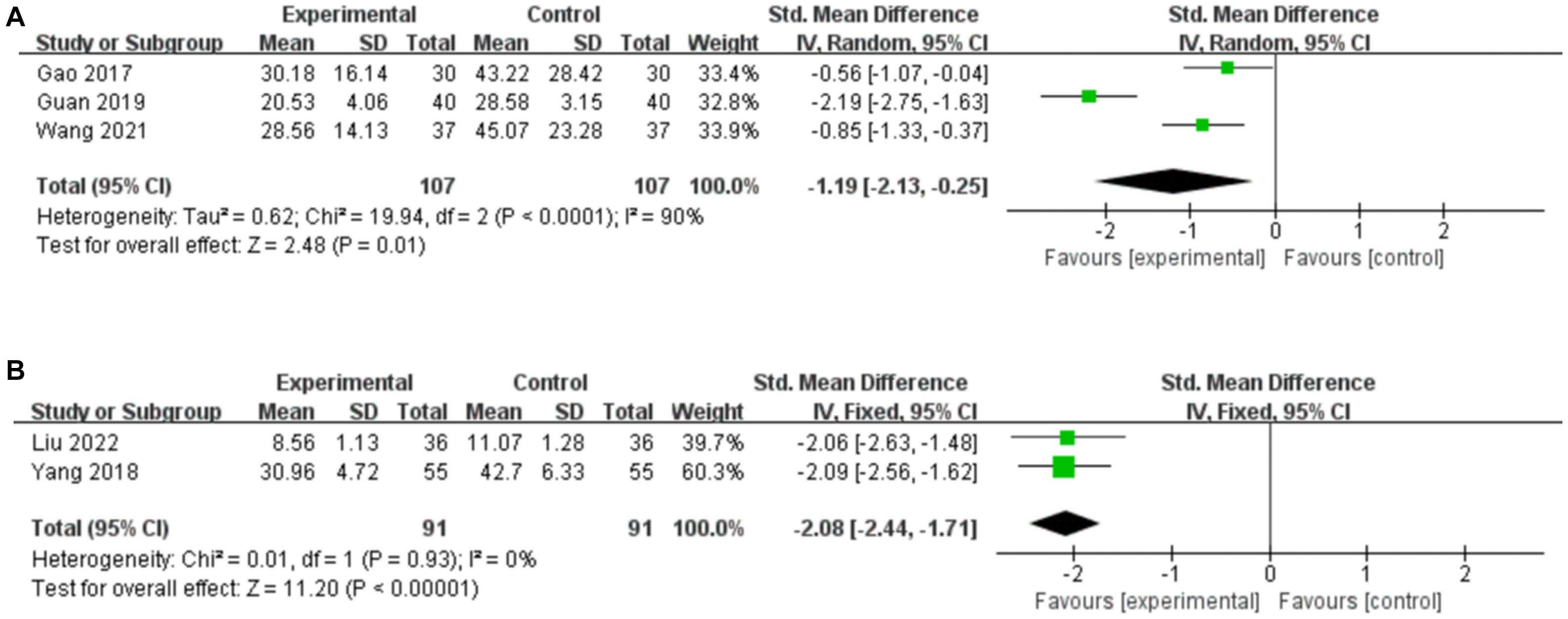
Forest plot of the time to first BM. **(A)** Acupuncture vs. CT. **(B)** Acupuncture plus CT vs. CT.

Three studies compared the time to first BM for acupuncture vs. CT, and the random-effects model was used because of the high heterogeneity among the studies (*p* < 0.0001, *I*^2^ = 90%). As shown in Figure 8A, acupuncture resulted in a greater reduction in the time to first BM than did CT (*SMD*: –1.19, 95% CI: −2.13 to −0.25, *p* = 0.01). Sensitivity analysis revealed that heterogeneity decreased significantly (*p* = 0.42, *I*^2^ = 0%) after removing the study by Guan (2019), which did not describe detailed methods for generating random sequences, thus leading to methodological heterogeneity.

##### 3.7.1.2. Acupuncture plus CT vs. CT

Two studies compared the time to first BM for acupuncture plus CT vs. CT, and the fixed-effects model was used because there was no heterogeneity among the studies (*p* = 0.93, *I*^2^ = 0%). As shown in Figure 8B, acupuncture plus CT resulted in a greater reduction in the time to first BM than did CT (*SMD*: –2.08, 95% CI: −2.44 to −1.71, *p* < 0.00001).

#### 3.7.2. Serum VIP levels

##### 3.7.2.1. Acupuncture vs. CT

**Figure 9:**
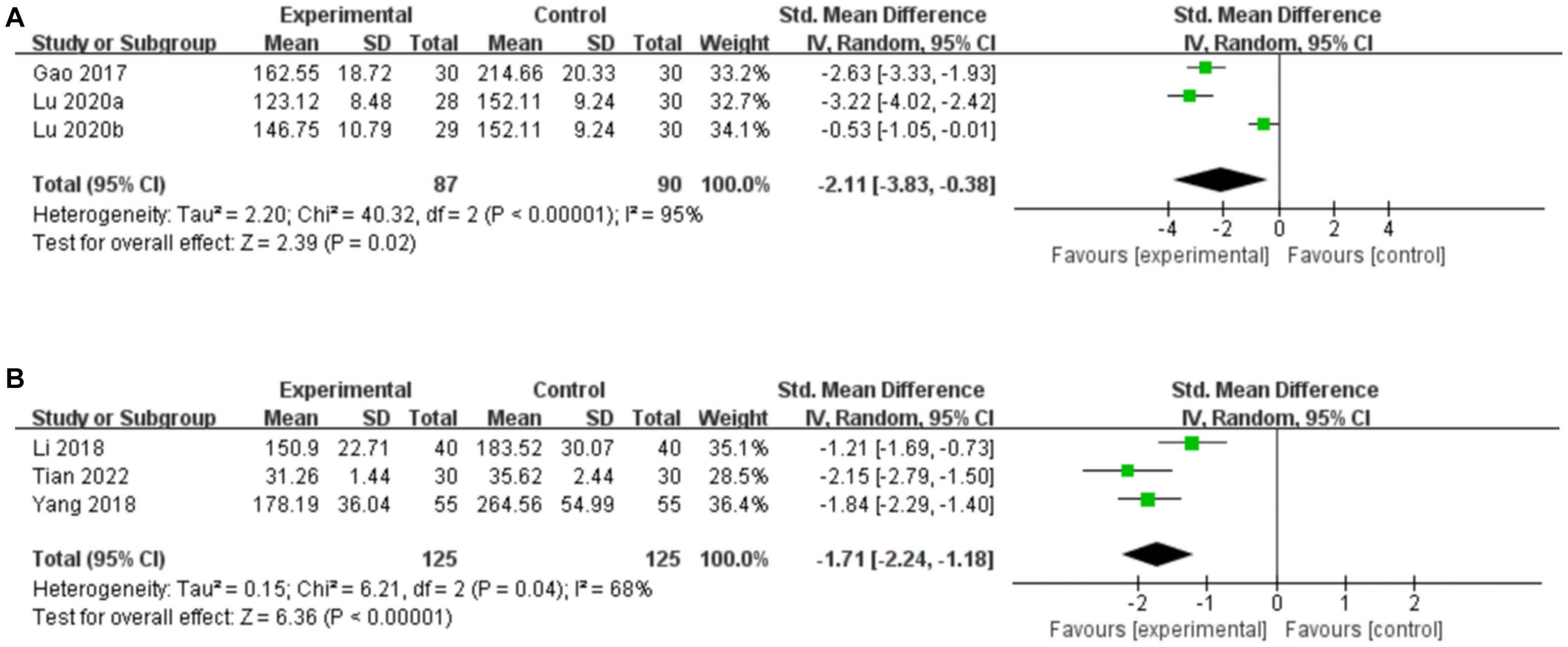
Forest plot of serum VIP levels. **(A)** Acupuncture vs. CT. **(B)** Acupuncture plus CT vs. CT.

Three studies compared serum VIP levels for acupuncture vs. CT, and the random-effects model was used because of the high heterogeneity among the studies (*p* < 0.00001, *I*^2^ = 95%). As shown in Figure 9A, acupuncture resulted in a greater reduction in serum VIP levels than did CT (*SMD*: –2.11, 95% CI: −3.83 to −0.38, *p* = 0.02). Sensitivity analysis revealed that heterogeneity decreased significantly (*p* = 0.28, *I*^2^ = 15%) after removing the study by Lu et al. (2020), which did not include the Tianshu acupoint, thus leading to clinical heterogeneity.

##### 3.7.2.2. Acupuncture plus CT vs. CT

Three studies compared serum VIP levels for acupuncture plus CT vs. CT, and the random-effects model was used because of the high heterogeneity among the studies (*p* = 0.04, *I*^2^ = 68%). As shown in Figure 9B, acupuncture plus CT resulted in a greater reduction in the serum level of VIP compared to CT (*SMD*: -1.71, 95% CI: −2.24 to −1.18, *p* < 0.00001). Sensitivity analysis demonstrated that the heterogeneity was significantly decreased (*p* = 0.45, *I*^2^ = 0%) after removing the studies by Li et al. (2018).

#### 3.7.3. Serum SP levels

##### 3.7.3.1. Acupuncture vs. CT

**Figure 10:**
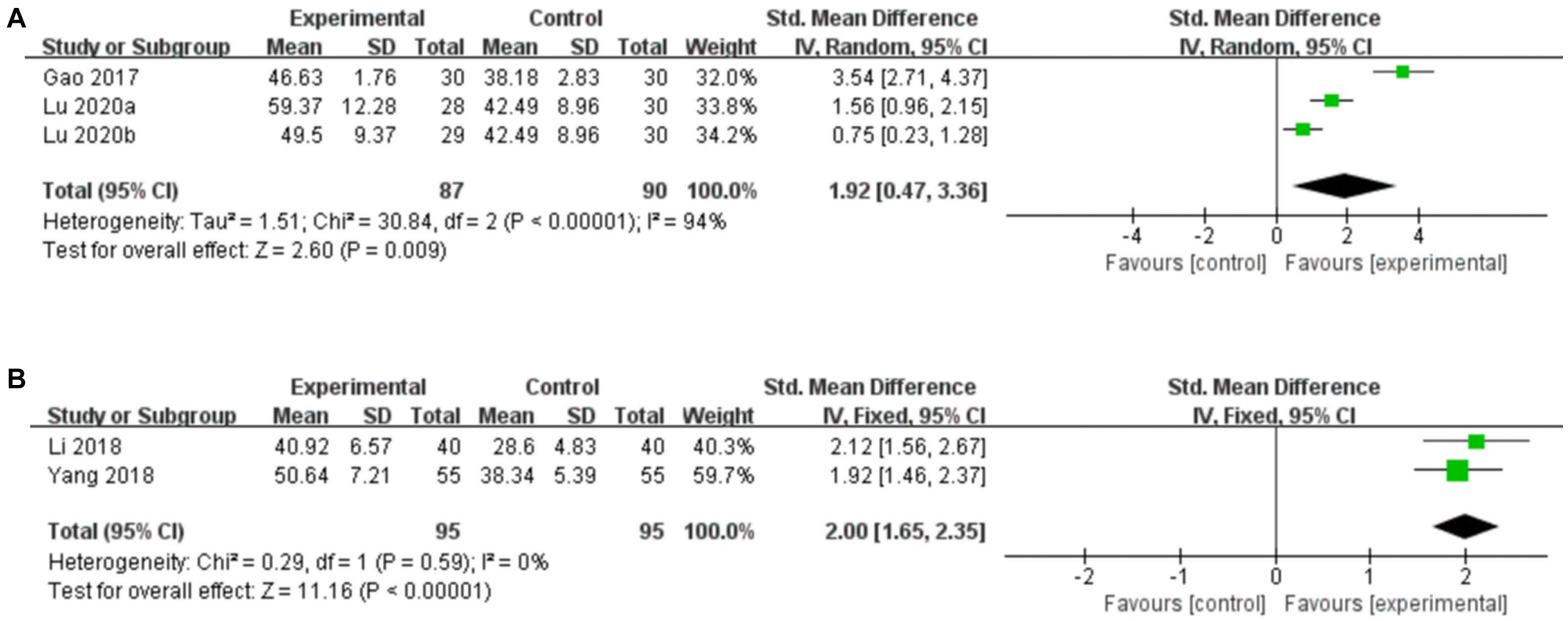
Forest plot of serum SP levels. **(A)** Acupuncture vs. CT. **(B)** Acupuncture plus CT vs. CT.

Three studies compared serum SP levels for acupuncture vs. CT, and the random-effects model was used because of the high heterogeneity among the studies (*p* < 0.00001, *I*^2^ = 94%). As shown in Figure 10A, we found that acupuncture was superior to CT in increasing serum levels of SP (*SMD*: 1.92, 95% CI: 0.47–3.36, *p* = 0.009). However, the sensitivity analysis demonstrated that the result for serum SP level was no longer significant (*SMD*: 2.13, 95% CI: −0.60 to 4.86, *p* = 0.13) after removing the study by Lu et al. (2020), suggesting that the conclusion is not stable.

##### 3.7.3.2. Acupuncture plus CT vs. CT

Two studies compared serum SP levels for acupuncture plus CT vs. CT, and the random-effects model was used because there was no heterogeneity among the studies (*p* = 0.59, *I*^2^ = 0%). As shown in Figure 10B, we found that acupuncture plus CT was superior to CT in increasing serum SP levels (*SMD*: 2.00, 95% CI: 1.65–2.35, *p* < 0.00001).

#### 3.7.4. BSS score

##### 3.7.4.1. Acupuncture vs. CT

**Figure 11:**
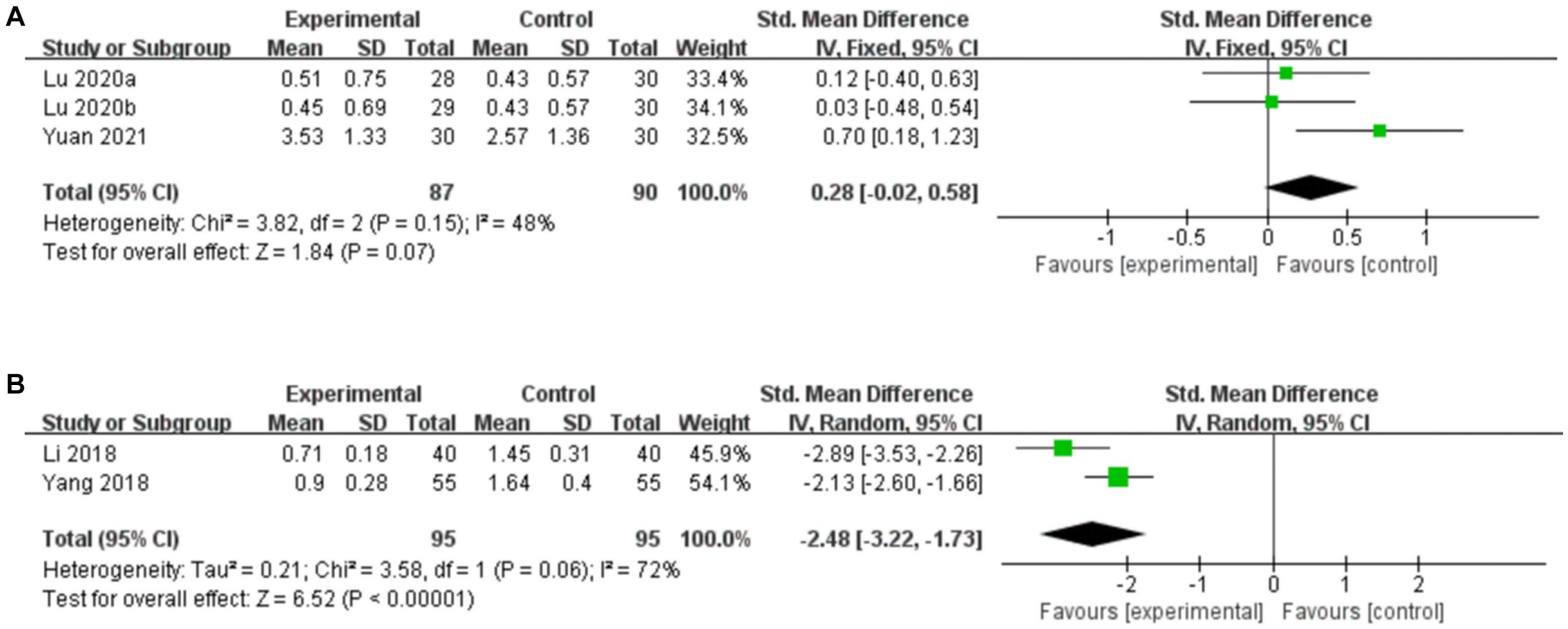
Forest plot of BSS score. **(A)** Acupuncture vs. CT. **(B)** Acupuncture plus CT vs. CT.

Three studies compared the BSS score for acupuncture vs. CT, and the fixed-effects model was used because of the low heterogeneity among the studies (*p* = 0.15, *I*^2^ = 48%). As shown in Figure 11A, acupuncture did not result in a greater reduction in the BSS score compared to CT (*SMD*: 0.28, 95% CI: −0.02 to 0.58, *p* = 0.07). Sensitivity analysis revealed that heterogeneity decreased significantly (*p* = 0.81, *I*^2^ = 0%) after removing the study by Yuan et al. (2021), which used the diagnostic criteria of Rome III, leading to clinical heterogeneity.

##### 3.7.4.2. Acupuncture plus CT vs. CT

Two studies compared the BSS score for acupuncture plus CT vs. CT, and the random-effects model was used because of the high heterogeneity among the studies (*p* = 0.06, *I*^2^ = 72%). As shown in Figure 11B, acupuncture plus CT resulted in a greater reduction in the BSS score than did CT (*SMD*: –2.48, 95% CI: −3.22 to −1.73, *p* < 0.00001).

#### 3.7.5. Adverse events

Thirteen studies reported AEs, of which four (Zhang et al., 2015; Gao et al., 2017; Liu Q. et al., 2018; Liu Z. et al., 2018; Liu and Wang, 2022) reported no AEs in either the experimental or control group. The primary AEs in the medication group were abdominal pain, diarrhea, nausea, vomiting, and abdominal bloating, while the primary AEs in the acupuncture treatment group were fainting during acupuncture.

##### 3.7.5.1. Acupuncture vs. CT

**Figure 12:**
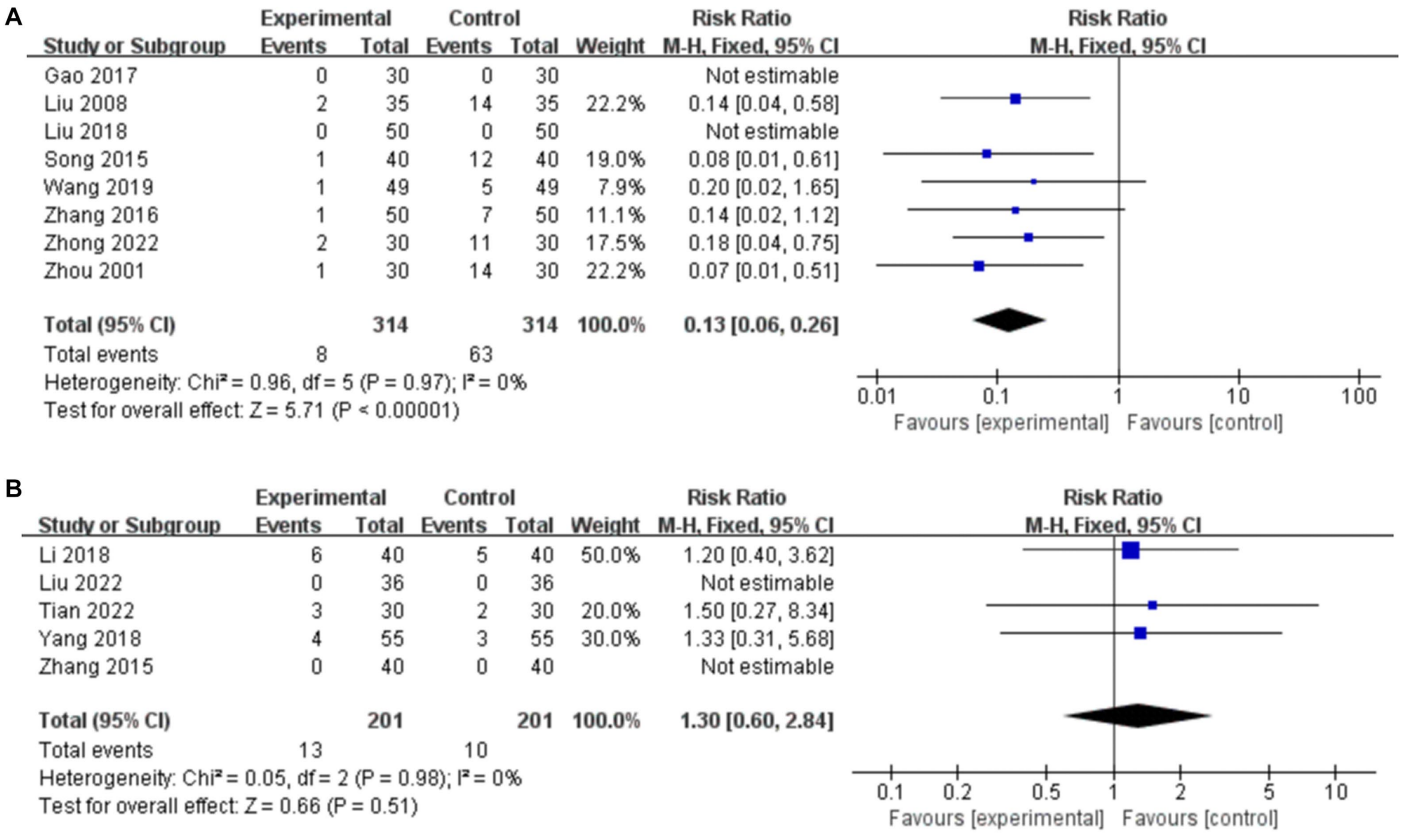
Forest plot of the incidence of AEs. **(A)** Acupuncture vs. CT. **(B)** Acupuncture plus CT vs. CT.

Eight studies compared AEs for acupuncture vs. CT, and the fixed-effects model was used because there was no heterogeneity among the studies (*p* = 0.97, *I*^2^ = 0%). As shown in Figure 12A, acupuncture resulted in a greater reduction in the incidence of AEs compared to CT (*RR*: 0.13, 95% CI: 0.06–0.26, *p* < 0.00001), and sensitivity analysis revealed the robustness of the conclusions (Supplementary Figure S2D).

##### 3.7.5.2. Acupuncture plus CT vs. CT

Five studies compared AEs for acupuncture plus CT vs. CT, and the fixed-effects model was used because there was no heterogeneity among the studies (*p* = 0.98, *I*^2^ = 0%). As shown in Figure 12B, acupuncture plus CT did not result in a greater reduction in the incidence of AEs than did CT (*RR*: 1.30, 95% CI: 0.60–2.84, *p* = 0.51), and sensitivity analysis revealed the robustness of the conclusions (Supplementary Figure S2E).

### 3.8. Risk of publication bias

**Figure 13:**
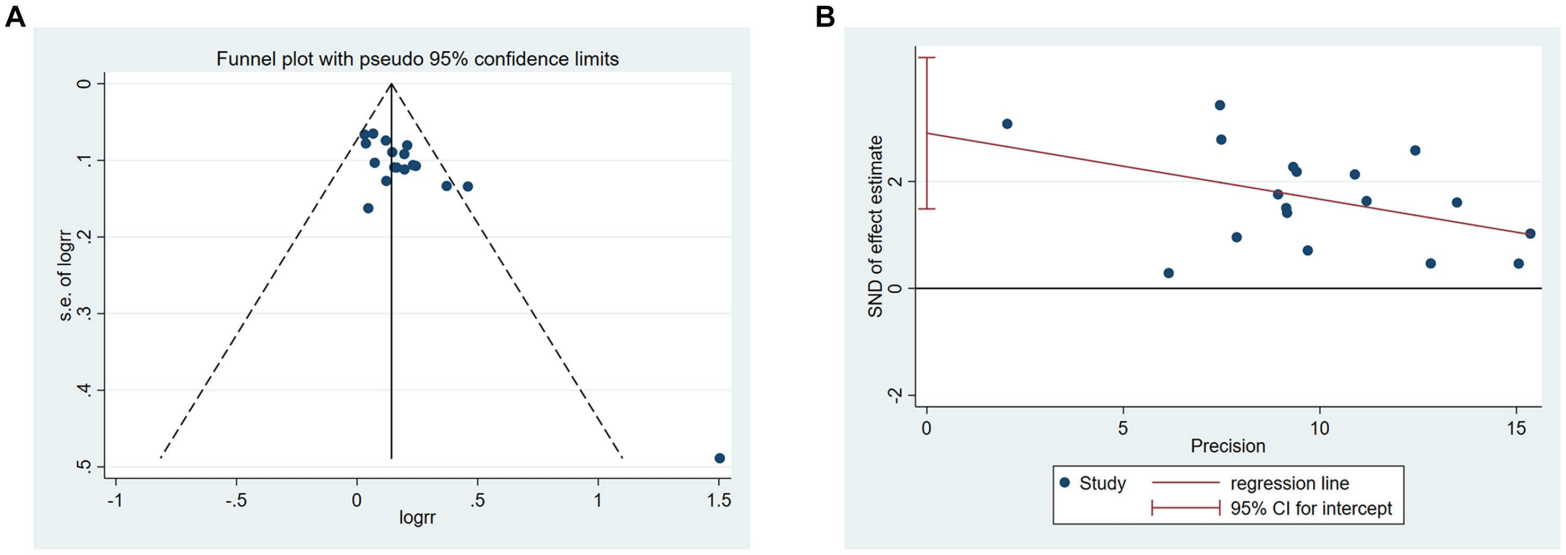
Publication bias of acupuncture vs. CT in the total responder rate. **(A)** Funnel plots. **(B)** Egger’s test.

The plot of acupuncture vs. CT on total responder rate was visibly asymmetric (Figure 13A), and Egger’s test revealed potential publication bias (Egger’s test *p* = 0.001) (Figure 13B). We conducted trim-and-fill test analysis to assess the effect of publication bias on the interpretation of the results, and we found that this publication bias did not affect the estimates, although several RCTs showing negative findings remained unpublished (Supplementary Table S4).

### 3.9. Certainty assessment

The certainty of evidence for the meta-analysis was evaluated by GRADE. The quality of evidence ranged from very low to high (Table 2). The primary reasons for downgrading were inconsistency (high heterogeneity) and imprecision (small sample size).

**Table 2:** GRADE summary of outcomes.

No.	Study design	Certainty assessment					Summary of results						Importance
		Risk of bias	Inconsistency	Indirectness	Imprecision	Other considerations	No of patients		Effect (95% CI)		Certainty		
							T	C	Relative	Absolute			
**Acupuncture vs. CT on total responder rate**													
19	RCT	Not Serious^a^	Serious^b^	Not serious	Not serious	Serious^f^	609	502	*RR* 1.16 (1.09 to 1.25)	–	⊕⊕○ ○	Low	Critical
**Acupuncture plus CT vs. CT on total responder rate**													
9	RCT	Not Serious^a^	Not Serious	Not Serious	Serious^d3^	Not serious^g^	309	248	*RR* 1.26 (1.17 to 1.35)	–	⊕⊕⊕ ○	Moderate	Critical
**Acupuncture vs. CT on constipation symptom score**													
7	RCT	Not serious ^a^	Not serious	Not serious	Not serious	Not serious ^g^	244	247	–	*SMD* 0.65 lower (0.83 lower to 0.46 lower)	⊕⊕ ⊕⊕	High	Critical
**Acupuncture vs. CT on time to first BM**													
3	RCT	Not serious^a^	Very serious^c^	Not serious	Serious^e^	Not serious^g^	107	107	–	*SMD* 1.19 lower (2.13 lower to 0.25 lower)	⊕○ ○ ○	Very low	Important
**Acupuncture plus CT vs. CT on time to first BM**													
2	RCT	Not serious^a^	Not serious	Not serious	Serious^e^	Not serious^g^	91	91	–	*SMD* 2.08 lower (2.44 lower to 1.71 lower)	⊕⊕ ⊕ ○	Moderate	Important
**Acupuncture vs. CT on serum VIP levels**													
3	RCT	Not serious^a^	Very serious^c^	Not serious	Serious^e^	Not serious^g^	87	90	–	*SMD* 2.11 lower (3.83 lower to 0.38 lower)	⊕○ ○ ○	Very low	Important
**Acupuncture plus CT vs. CT on serum VIP levels**													
3	RCT	Not serious^a^	Serious ^b^	Not serious	Serious ^e^	Not serious ^g^	125	125	–	*SMD* 1.71 lower (2.24 lower to 1.18 lower)	⊕⊕ ○ ○	Low	Important
**Acupuncture vs. CT on serum SP levels**													
3	RCT	Not serious^a^	Very serious^c^	Not serious	Serious^e^	Not serious^g^	87	90	–	*SMD* 1.92 higher (0.47 higher to 3.36 higher)	⊕○ ○ ○	Very low	Important
**Acupuncture plus CT vs. CT on serum SP levels**													
2	RCT	Not serious ^a^	Not serious	Not serious	Serious^e^	Not serious^g^	95	95	–	*SMD* 2.00 higher (1.65 higher to 2.35 higher)	⊕⊕ ⊕ ○	Moderate	Important
**Acupuncture vs. CT on BSS score**													
3	RCT	Not serious^a^	Not serious	Not serious	Very serious ^d1 + e^	Not serious^g^	87	90	–	*SMD* 0.28 higher (0.02 lower to 0.58 higher)	⊕⊕ ○ ○	Low	Important
**Acupuncture plus CT vs. CT on BSS score**													
2	RCT	Not serious^a^	Serious^b^	Not serious	Serious^e^	Not serious^g^	95	95	–	*SMD* 2.48 lower (3.22 lower to 1.73 lower)	⊕⊕ ○ ○	Low	Important
**Acupuncture vs. CT on adverse events**													
8	RCT	Not serious^a^	Not serious	Not serious	Serious^d3^	Not serious^g^	314	314	*RR* 0.13 (0.06 to 0.26)	–	⊕⊕ ⊕ ○	Moderate	Important
**Acupuncture plus CT vs. CT on adverse events**													
5	RCT	Not serious^a^	Not serious	Not serious	Serious ^d2 + d3^	Not serious ^g^	201	201	*RR* 1.30 (0.60–2.84)	–	⊕⊕ ⊕ ○	Moderate	Important

C, control group; CI, confidence interval; RCT, randomized controlled trial; *RR*, risk ratio; *SMD*, standardized mean difference; T, treatment group. ^a^Most of the included studies were at unclear or low risk of bias; ^b^50% < *I*^2^ < 75% for heterogeneity; ^c^*I*^2^ ≥ 75% for heterogeneity; ^d1^95% CI contains 0; ^d2^95% CI contains 1; ^d3^*RR* < 0.75 or > 1.25; ^e^Small sample size; ^f^Publication bias; ^g^No test for publication bias.

## 4. Discussion

### 4.1. Summary of main findings

In this study, a total of 30 RCTs involving 2,220 (1,125/1,095) patients were included to systematically evaluate the efficacy and safety of acupuncture treatments for PSC. We found that both acupuncture and acupuncture plus CT offer significant benefits over CT on metrics of total responder rate, serum SP levels, time to first BM, and serum VIP levels. Acupuncture was also superior to CT in reducing constipation symptom scores, but the level of evidence was very low to high because of potential publication bias and heterogeneity. Acupuncture operates by multiple mechanisms.

Acupuncture operates by multiple mechanisms. Clinical studies (Xiong et al., 2014; Chen et al., 2022) demonstrate that acupuncture regulates autonomic function by increasing vagal activity and inhibiting sympathetic activity, thereby affecting the central nervous system and reflex pathways involved in defecation. The treatment rebalances inhibitory and excitatory gastrointestinal hormone levels to promote colonic motility, shorten colonic transit time, and improve constipation symptoms. Xu et al. (2020) observed that in patients with functional constipation, compared with mosapride & sham EA group and mosapride control group, EA significantly increased the weekly spontaneous bowel movements, improved stool consistency, and reduced the intensity of defecating difficulty. Meanwhile, it also ameliorated the quality-of-life scores, and there were no serious adverse events during the course of the study. The potential mechanisms of acupuncture stimulation in promoting gastrointestinal function were studied more deeply in animal experiments. Studies have confirmed that acupuncture not only regulates the level of hormones related to intestinal motility (Zhu et al., 2016; Jang et al., 2017), but also improves the morphologic structure of colonic smooth muscle (He et al., 2023), and rebalances the gut microbiota (Xu et al., 2023). Wang et al. (2023) found that acupuncture could improve enteric glial cells autophagy by inhibiting PI3K/AKT/mTOR signaling. Moreover, acupuncture could restore gastrointestinal basic electrical rhythm by increasing the number of interstitial cells of Cajal (He et al., 2023).

Notably, the two interventions differed in the degree of improvements in BSS scores and AEs. Regarding BSS scores, acupuncture offered no significant advantage over CT, while acupuncture plus CT was superior to CT, which is consistent with previous findings (Zheng et al., 2018; Wang et al., 2020; Liu et al., 2021) and may result from direct action on the intestines that changes stool consistency to facilitate defecation (Chang et al., 2022, 2023). In contrast, acupuncture operates through a series of neurological and endocrine mechanisms to improve gastrointestinal tract function, and we found that acupuncture synergizes well with CT to effectively improve stool consistency, even though short-term treatment with acupuncture alone does not offer relief. Regarding safety, acupuncture alone was superior to CT, but there was no significant difference when comparing acupuncture plus CT vs. CT. It is clear that acupuncture does not cause serious AEs as a monotherapy or an adjunctive therapy.

### 4.2. Secondary findings

We evaluated the effects of treatment duration, acupuncture frequency, types of control interventions, and needle retention time on total responder rates, which are relevant to clinical practice. We found that acupuncture significantly improves total responder rate with all studied treatment frequencies, control interventions, and defined needle retention times, and is effective as a monotherapy or an adjunctive therapy. Notably, though, acupuncture therapy was not found effective with uncertain needle retention time. Moreover, subgroup meta-analysis revealed that treatment durations of >2 weeks may be more effective than shorter treatment regiments at improving total responder rate. Furthermore, we evaluated acupuncture treatment regimens using STRICTA and summarized commonly used acupoints, providing scientific guidance for clinical practice and for the design and implementation of clinical studies. A total of 51 acupoints with frequencies ranging from 1 to 23 were used; the most commonly used acupoints for PSC were Tianshu (ST25), Zusanli (ST36), Qihai (RN6), and Zhigou (SJ6).

Because of the high heterogeneity of meta-analysis results, we performed sensitivity analysis and subgroup analysis to explore and eliminate sources of heterogeneity. We found that heterogeneity in total responder rates resulted from the inconsistency of treatment duration, while the heterogeneity in serum VIP levels resulted from differences in therapeutic acupoints. The source of heterogeneity in the time to first BM resulted from the higher risk of overall bias in the included studies, and the source of heterogeneity in the BSS score resulted from differences in diagnostic criteria. Some outcome indicators were still highly heterogeneous after our corrections, meaning that additional studies are needed to determine if acupuncture plus CT can improve serum VIP levels and if acupuncture can increase serum SP levels.

### 4.3. Strengths compared to previous studies

Compared to previous studies, our study has several strengths. First, we found that treatment duration may influence efficacy, thus providing a reference for clinical practice and clinical research. Second, we have adopted updated and objective constipation-related indicators such as time to first BM and serum gastrointestinal peptide levels to evaluate the effects of acupuncture, further supporting the use of acupuncture in treating PSC. Third, we enhanced the credibility of our results by using sensitivity analyses and subgroup analyses to explore the sources of heterogeneity, the robustness of the results, and the effects of some characteristics on the efficacy of acupuncture. Fourth, the STRICTA checklist and GRADE were used to assess clinical trial reporting quality and evidence quality, respectively. Finally, to minimize heterogeneity in the included studies, we limited the types of control interventions used and we excluded co-treatment with herbal formulas, Chinese patent medicines, and acupressure.

### 4.4. Limitations

Some limitations in this work should be noted. First, there are no standardized diagnostic criteria for PSC, and the different Rome diagnostic criteria (e.g., Rome II vs. Rome IV) may affect the assessment of acupuncture efficacy. Second, all included studies were conducted in China, and the conclusions of our study must be verified in patients of other races. Third, the strength of our conclusions may be limited by small sample sizes, poor methodological quality (e.g., some of the included studies did not describe the randomization methodology and allocation concealment in detail), and the potential risk of bias. Fourth, most of the included studies do not report complete acupuncture details according to the STRICTA checklist, which may lead to bias in the interpretation of results. Fifth, Differences in gender and age of patients in RCTs, as well as in interventions in the control group, may lead to a risk of bias that reduces the credibility of the findings, so we should be cautious about the conclusions. Finally, we were unable to compare acupuncture vs. sham acupuncture, and the long-term effects of acupuncture, because of the limited number of studies and follow-up data.

### 4.5. Implications for future research

Our meta-analysis suggests that acupuncture has great potential for the treatment of PSC and deserves further exploration. First, we found that 2 weeks may be the minimum effective treatment duration for acupuncture efficacy, though more research is needed to verify the robustness and scientific validity of this conclusion, as high-quality evidence is limited. Second, there are still few PSC trials that use sham acupuncture as a control treatment to eliminate the non-specific effects of acupuncture; future clinical studies should use sham acupuncture as a control. Third, the quality of future studies would be improved by standardizing study protocols, unifying diagnostic criteria, and improving the implementation of randomization, allocation concealment, and blinding. In addition, more scientific and objective outcome indicators such as complete spontaneous BMs and recurrence rates should be selected, and follow-up times should be extended. Finally, clinical studies should strictly follow the CONSORT 2010 statement (Schulz et al., 2010) and STRICTA (MacPherson et al., 2010) to improve reporting quality, especially regarding details of acupuncture treatments that are critical for the accuracy of results. More reliable guidance for clinical practice would be provided by large, high-quality, multicenter, double-blinded RCTs that comprehensively assess the efficacy and safety of acupuncture and identify efficient and rational treatment protocols.

## 5. Conclusion

Acupuncture alone or as an adjunctive treatment for PSC is superior to CT in terms of efficacy and safety, indicating that acupuncture is a potential alternative therapy for PSC. However, because the evidence quality in this study is unstable, more well-designed long-term follow-up RCTs are needed to evaluate acupuncture efficacy and safety.

## Data availability statement

The original contributions presented in the study are included in the article/Supplementary material, further inquiries can be directed to the corresponding author.

## Author contributions

TS: Conceptualization, Data curation, Formal analysis, Investigation, Project administration, Resources, Validation, Visualization, Writing – original draft. KW: Data curation, Formal analysis, Investigation, Resources, Validation, Visualization, Writing – original draft. LL: Data curation, Investigation, Resources, Writing – original draft. MY: Data curation, Investigation, Writing – original draft. LZ: Data curation, Investigation, Writing – original draft. MZ: Data curation, Investigation, Writing – original draft. SY: Data curation, Investigation, Writing – original draft. JW: Data curation, Investigation, Writing – original draft. JL: Conceptualization, Funding acquisition, Project administration, Supervision, Writing – review & editing.
