# Supplementary material for: Efficacy and safety of acupuncture in post-stroke constipation: a systematic review and meta-analysis
Source: Front Neurosci. 2023 Sep 26;17:1275452. doi: 10.3389/fnins.2023.1275452 (PMC10562632; doi:10.3389/fnins.2023.1275452)
Supplement: Supplementary file 1 [file Data_Sheet_1.docx]

Supplementary Material

**Table of contents**

[1 Supplementary Tables 1](#_Toc23377)

[Supplementary Table 1. Reasons for exclusion of studies after full text review. 1](#_Toc5260)

[Supplementary Table 2. Frequency ranking of acupoints. 9](#_Toc11974)

[Supplementary Table 3. STRICTA checklist for the included studies. 10](#_Toc29963)

[Supplementary Table 4. Trim-and-fill test of total responder rate in comparison of acupuncture vs. CT. 13](#_Toc28501)

[2 Supplementary Figures 14](#_Toc13658)

[Supplementary Figure 1. Reporting details of STRICTA checklist. 14](#_Toc12302)

[Supplementary Figure 2. Sensitivity analysis. 15](#_Toc22359)

[3 Supplementary File 16](#_Toc25151)

[Supplementary File 1. Search strategy. 16](#_Toc10867)

[Supplementary File 2. The PRISMA checklist of this meta-analysis. 30](#_Toc5504)

# Supplementary Tables

## Supplementary Table 1. Reasons for exclusion of studies after full text review.

| Exclusion reasons （n） | Study ID | Title of excluded studies |
| --- | --- | --- |
| The intervention did not meet the inclusion criteria (n = 93) | Huang 2022 | Exploring the clinical efficacy of qi cross moxibustion combined with primary care in the treatment of qi stagnation type constipation after stroke based on the brain-gut axis |
|  | Ding 2022 | Clinical efficacy of combined Chinese and Western medicine in the treatment of post-stroke constipation |
|  | Chen 2022 | Clinical Observations on Bilateral Acupuncture for Constipation in Elderly Stroke Recovery |
|  | Cai 2022 | Traditional Chinese medicine nursing techniques and effect observation for elderly patients with constipation after stroke |
|  | Su 2022 | Study on the rehabilitation effect of traditional Chinese medicine nursing techniques on gastrointestinal dysfunction in elderly stroke patients |
|  | Li 2021 | Observation on the effect of spreading moxibustion in the acupoint area of Da Huang Shen Que on patients with constipation during the recovery period of stroke |
|  | Guo 2021 | Observation on the application effect of Chinese medicine nursing program in patients with constipation during the recovery period of cerebral infarction |
|  | Shi 2021 | Clinical observation of qi cross moxibustion in the treatment of constipation of qi deficiency and blood stasis type after stroke |
|  | Li 2021 | The effect of San Jiao Tongzhi method on the quality of life of patients with post-stroke constipation and gas deficiency and blood stasis syndrome |
|  | Bu 2021 | Effects of combined Chinese and Western medicine nursing intervention on stroke patients with constipation |
|  | Zhang 2021 | Analysis of the effect of Chinese medicine nursing on improving constipation symptoms in stroke patients |
|  | He 2021 | Observation on the effect of traditional Chinese medicine in caring for constipation in elderly stroke patients |
|  | Tang 2021 | Evaluation of the intervention effect of Chinese medicine nursing on constipation in patients with stroke |
|  | Xu 2021 | A study on the use of Chinese medicine nursing program in patients with constipation after recovery from cerebral infarction |
|  | Wei 2021 | Study on the efficacy of cake moxibustion in the treatment of post-stroke constipation |
|  | Wang 2021 | The experience of traditional Chinese medicine care for patients with constipation after cerebral stroke |
|  | Zhang 2021 | Analysis of the effect of combined Chinese and Western medicine care for constipation in patients with cerebral stroke |
|  | Liang 2020 | Therapeutic efficacy of fire-needle acupuncture with three intestinal needles in the treatment of constipation with yang deficiency after stroke |
|  | Wang 2020 | Observations on the application of traditional Chinese medicine nursing in female elderly post-stroke constipation patients |
|  | Wang 2020 | Observation on the application effect of Chinese medicine characteristic nursing in patients with constipation after stroke |
|  | Xing 2020 | Exploring the clinical efficacy of acupuncture in the treatment of constipation after ischemic stroke |
|  | Dou 2020 | Observation on the effect of Chinese medicine integrated therapy on postoperative constipation in patients with cerebral hemorrhage |
|  | Xie 2020 | Nursing experience of Chinese medicine characteristic nursing intervention for constipation in patients with cerebral infarction |
|  | Wang 2020 | Analysis of the causes of constipation in stroke patients and traditional Chinese medicine care methods |
|  | Li 2020 | The effect of early Chinese medicine nursing intervention on the maintenance of gastrointestinal function in stroke patients |
|  | Wen 2020 | Analysis of the effect of combined Chinese and Western medicine care for constipation in patients with cerebral stroke |
|  | Liang 2020 | Analysis of the clinical implementation effect of Chinese medicine nursing intervention in patients with post-stroke constipation |
|  | Li 2020 | Analysis of the causes of constipation in bedridden patients with stroke and nursing countermeasures |
|  | Ma 2019 | Evaluation of the role of Chinese medicine nursing program intervention in promoting the recovery of defecation function in patients with post-stroke constipation |
|  | Weng 2019 | Clinical implementation effect of Chinese medicine nursing intervention in patients with post-stroke constipation |
|  | Hao 2019 | Observation and analysis of the effect of traditional Chinese medicine nursing combined with conventional neurology nursing intervention in patients with cerebral infarction constipation |
|  | Wen 2019 | Analysis of identification of constipation and nursing countermeasures in patients with stroke |
|  | Zhao 2019 | Analysis of the improvement effect of traditional Chinese medicine nursing on constipation in elderly stroke patients |
|  | Wang 2019 | Observation and analysis of the effect of traditional Chinese medicine nursing combined with conventional neurology nursing intervention in patients with cerebral infarction constipation |
|  | Ma 2019 | Nursing construction of constipation in 95 patients with cerebral infarction |
|  | Zheng 2019 | Analysis of the effect of traditional Chinese medicine nursing on constipation in elderly stroke patients |
|  | Wang 2019 | Comparison of the effects of different nursing interventions in patients with constipation after stroke Key Points |
|  | Feng 2019 | Effect of nursing intervention on constipation and electrolyte disorders in patients with cerebral hemorrhage |
|  | Zhou 2018 | Observation on the clinical implementation effect of Chinese medicine nursing intervention in patients with post-stroke constipation |
|  | Sun 2018 | Analysis of the effect of Chinese medicine nursing intervention on constipation in patients with stroke |
|  | Lin 2018 | The role of Chinese medicine nursing program intervention in promoting the recovery of defecation function in patients with post-stroke constipation |
|  | Zhou 2018 | Clinical Observation on Chinese Medicine Comprehensive Nursing Treatment of Stroke Constipation |
|  | Xu 2018 | Clinical observation of umbilical moxibustion in the treatment of constipation in stroke disease |
|  | Song 2018 | Application of Chinese medicine nursing in patients with constipation during recovery from cerebral infarction |
|  | Jin 2018 | Application of Traditional Chinese Medicine Nursing Care in Elderly Patients with Constipation after Stroke |
|  | Liu 2018 | Observation on the effect of traditional Chinese medicine in caring for constipation in elderly stroke patients |
|  | Yang 2018 | Analysis of the effect of Chinese medicine nursing on constipation in elderly stroke patients |
|  | Sun 2018 | Analysis of Traditional Chinese Medicine Nursing Intervention for Patients with Stroke Constipation |
|  | Yin 2018 | Observation on the effect of traditional Chinese medicine nursing combined with conventional neurology nursing intervention in patients with cerebral infarction constipation |
|  | Peng 2018 | Impact of nursing intervention in patients with cerebral hemorrhage constipation |
|  | Sun 2017 | Observation on the effect of traditional Chinese medicine in caring for constipation in elderly stroke patients |
|  | Yin 2017 | The effect of Chinese medicine comprehensive care on the quality of life of patients with stroke constipation |
|  | Lu 2017 | To study and analyze the effect of using combined Chinese and Western medicine care for patients with constipation in cerebral stroke |
|  | Chen 2017 | Observation on the effect of traditional Chinese medicine in caring for constipation in elderly stroke patients |
|  | Zhao 2016 | Chinese medicine nursing experience of constipation in 60 cases of stroke patients |
|  | Xu 2016 | Observation on the effect of traditional Chinese medicine nursing intervention on constipation in stroke patients |
|  | Deng 2016 | Observation on the effect of Chinese medicine nursing intervention on constipation in patients with stroke |
|  | Tang 2016 | Clinical effects of mild moxibustion at Shenque point on promoting defecation function in bedridden patients with post-stroke sequelae |
|  | Xu 2016 | Therapeutic efficacy observation of traditional Chinese medicine evidence-based care for constipation in patients with stroke |
|  | Xu 2016 | Analysis of the effect of traditional Chinese medicine on the care of constipation in elderly patients with stroke |
|  | He 2016 | Exploration of the efficacy of acupuncture therapy in Chinese medicine on constipation in stroke |
|  | Hou 2016 | Application of evidence-based nursing care in the treatment of stroke patients with constipation by combining traditional Chinese and Western medicine |
|  | Chen 2016 | Nursing experience of constipation in stroke patients |
|  | Yu 2016 | Exploring the effect of early Chinese medicine nursing intervention on the maintenance of gastrointestinal function in stroke patients |
|  | Yang 2016 | Comprehensive nursing intervention in the prevention and treatment of constipation in stroke patients |
|  | Chen 2016 | Observation on the effect of nursing intervention on constipation in stroke patients |
|  | Zhou 2015 | Effects of moxibustion combined with traditional Chinese medicine evidence-based nursing intervention on patients with constipation in the acute phase of stroke |
|  | Liu 2015 | Efficacy of navel moxibustion in the treatment of post-stroke constipation |
|  | Liu 2015 | Analysis of the effect of using nursing intervention to improve patients' constipation in neurological stroke patients |
|  | Huang 2014 | Observation on the effect of comprehensive nursing intervention in patients with stroke complicated with constipation |
|  | Zhao 2014 | Observation on the clinical efficacy of Chinese medicine nursing intervention on stroke combined with constipation |
|  | Liao 2013 | Effect and analysis of DAJ-5B type moxibustion therapeutic instrument on the incidence of constipation in patients with cerebral infarction |
|  | Sun 2013 | Analysis of the effect of combined Chinese and Western medicine care for constipation in patients with cerebral stroke |
|  | Wang 2013 | Clinical efficacy analysis of multifaceted treatment of constipation after stroke |
|  | Li 2012 | Traditional Chinese medicine care for constipation in stroke patients |
|  | Du 2012 | The effect of systemic nursing intervention with acupoint stimulation on the efficacy of patients with stroke constipation |
|  | Lu 2012 | Traditional Chinese medicine care of constipation in patients with stroke and visceral solidity |
|  | Lan 2012 | Evaluation of the effect of applying traditional Chinese medicine evidence-based care to patients with post-stroke constipation |
|  | Wei 2011 | Clinical observation on 30 cases of constipation after stroke treated by acupuncture |
|  | Yang 2011 | Clinical observation on acupuncture treatment of 36 cases of constipation after cerebral infarction |
|  | Xu 2011 | Traditional Chinese medicine treatment and care of constipation in patients with hemorrhagic stroke |
|  | Xia 2011 | Traditional Chinese medicine nursing care for stroke patients with bedridden constipation |
|  | Wang 2011 | Observation on the effect of nursing intervention on constipation in hemiplegic patients with stroke |
|  | Meng 2011 | Observation on the effect of overall nursing intervention on stroke patients with constipation |
|  | Liu 2010 | The effect of comprehensive nursing intervention on patients with constipation in hemorrhagic stroke |
|  | Chen 2010 | The effect of anticipatory nursing care on constipation in traumatic subarachnoid hemorrhage |
|  | Yang 2009 | Clinical observation of constipation after cerebral infarction treated by electroacupuncture |
|  | Xiao 2009 | Clinical efficacy evaluation of detoxification and visceral method in treating constipation of phlegm-heat visceral solid type in the acute stage of ischemic stroke |
|  | Zhao 2008 | Nursing intervention effect observation of 53 cases of stroke patients with constipation |
|  | Tan 2007 | Thirty cases of constipation after cerebral infarction treated by acupuncture with the method of opening the bowels and its effect on neurological deficits |
|  | Liu 2007 | Treatment of hemorrhagic stroke with constipation by acupuncture with the method of opening the bowels and opening the orifices. |
|  | Hu 2006 | Acupuncture treatment of constipation after stroke: 40 cases |
|  | Cui 2004 | Observation on the efficacy of acupuncture in treating constipation in the recovery period of stroke |
| The design did not meet the inclusion criteria (n = 10) | Liu 2019 | Study on the clinical effect of Yu Mu Yuan point acupuncture on patients with post-stroke constipation |
|  | Huang 2016 | Evaluation of the clinical effect of warm acupuncture in the treatment of post-stroke deficiency constipation |
|  | Zhao 2016 | Clinical study of acupuncture in the treatment of post-stroke constipation |
|  | Xiao 2011 | Post-stroke constipation: Insights from traditional Chinese medicine diagnosis and treatment |
|  | Feng 2011 | Traditional Chinese medicine diagnostic care for constipation in stroke patients |
|  | Li 2010 | Head acupuncture with abdominal acupuncture for the treatment of post-stroke constipation in 50 cases |
|  | Qi 2010 | Traditional Chinese medicine care for constipation in stroke patients |
|  | Lu 2010 | Evidence-based nursing care in stroke patients with bedridden constipation |
|  | Zeng 2009 | Clinical care of 126 cases of stroke patients with constipation |
|  | Zhu 2007 | Chinese and western medical treatment and nursing care of 36 cases of constipation in patients with stroke |
| Not retrieved (n = 6) | Tang 2017 | Study on the effect of traditional Chinese medicine care for patients with constipation complicated by stroke |
|  | Cui 2016 | Analysis of the effect of combined Chinese and Western medicine care for constipation in patients with cerebral stroke |
|  | Li 2014 | Analysis of the effect of nursing intervention with acupoint stimulation on the efficacy of patients with stroke constipation |
|  | Tao 2013 | Study on the clinical effect of Chinese medicine nursing intervention on constipation in patients with stroke |
|  | Sun 2013 | Observation on the effect of nursing intervention on the prevention and treatment of constipation in patients with cerebral hemorrhage |
|  | Cui 2004 | Clinical observation on electroacupuncture treatment of constipation in acute stage of cerebral hemorrhage in 30 cases |
| The outcome did not meet the inclusion criteria (n = 5) | Li 2021 | Clinical observation of acupuncture combined with prucalopride succinate tablets in the treatment of post-stroke constipation |
|  | Wen 2018 | Treatment of constipation after ischemic stroke with Wang's "old ten needles": 40 cases |
|  | Gan 2017 | Clinical efficacy analysis of warm acupuncture in the treatment of post-stroke constipation |
|  | Wang 2017 | Clinical observation on electroacupuncture treatment of post-stroke constipation |
|  | Wu 2014 | Clinical observation on the treatment of post-stroke constipation by head acupuncture with warm acupuncture and moxibustion at Tianshu and Guanyuan points |

## Supplementary Table 2. Frequency ranking of acupoints.

| acupoint | frequency | acupoint | frequency | acupoint | frequency |
| --- | --- | --- | --- | --- | --- |
| ST25 | 23 | BL20 | 3 | Qiwaihuan | 2 |
| ST36 | 8 | ST29 | 3 | Qineihuan | 1 |
| RN6 | 8 | ST40 | 3 | Qizheng | 1 |
| SJ6 | 8 | RN10 | 2 | Qimen | 1 |
| RN12 | 6 | KI6 | 2 | Qijiao | 1 |
| LR3 | 6 | BL23 | 2 | LI6 | 1 |
| ST37 | 6 | RN8 | 2 | LU7 | 1 |
| PC6 | 5 | SP6 | 2 | EX-LE14 | 1 |
| RN17 | 5 | LI4 | 2 | LU6 | 1 |
| RN4 | 5 | EX-HN3 | 1 | BL18 | 1 |
| SP4 | 4 | BL15 | 1 | SP14 | 1 |
| BL25 | 4 | BP-UE3 | 1 | BL13 | 1 |
| ST28 | 4 | KI3 | 1 | EX-LE11 | 1 |
| foot movement sensory area | 4 | SP3 | 1 | MS5 | 1 |
| ST39 | 3 | EX-HN1 | 1 | SP15 | 1 |
| Waishuidao | 3 | RN5 | 1 | LU5 | 1 |
| Waiguilai | 3 | DU24 | 1 | DU20 | 1 |

## Supplementary Table 3. STRICTA checklist for the included studies.

| **Author (Year)** | **1 Acupuncture rationale** | | | **2 Details of needling** | | | | | | | **3 Treatment regimen** | | **4 Complementary interventions** | | **5 Practitioner background** | **6 Control or comparator interventions** | |
| --- | --- | --- | --- | --- | --- | --- | --- | --- | --- | --- | --- | --- | --- | --- | --- | --- | --- |
|  | **1a** | **1b** | **1c** | **2a** | **2b** | **2c** | **2d** | **2e** | **2f** | **2g** | **3a** | **3b** | **4a** | **4b** | **5** | **6a** | **6b** |
| Zhong et al., (2022) | Y | Y | N | Y | Y | Y | Y | Y | Y | Y | Y | Y | N | N | N | Y | Y |
| Yuan et al., (2021) | Y | Y | N | N | Y | Y | Y | Y | Y | N | Y | Y | Y | N | N | Y | Y |
| Wang et al., (2021) | Y | Y | Y | N | Y | Y | Y | Y | Y | Y | Y | Y | N | N | N | N | Y |
| Lu et al., (2020) | Y | Y | N | Y | Y | Y | Y | Y | Y | Y | Y | Y | N | N | N | Y | Y |
| Guan et al., (2019) | Y | Y | N | N | Y | N | N | N | Y | N | Y | Y | N | N | N | N | N |
| Wang et al., (2019) | Y | Y | N | Y | Y | N | Y | Y | Y | Y | Y | Y | N | N | N | N | Y |
| Luo, (2019) | Y | Y | N | Y | Y | Y | Y | Y | Y | Y | Y | Y | N | N | N | N | Y |
| Liu et al., (2018) | Y | Y | N | N | Ya | Y | Y | N | Y | Y | Y | Y | N | N | N | N | Y |
| Gao et al., (2017) | Y | Y | N | N | Ya | Y | Y | N | Y | Y | Y | Y | N | N | N | Y | Y |
| Zhang, (2016) | Y | N | N | N | Y | N | Y | Y | Y | Y | Y | Y | Y | N | N | N | Y |
| Song and Liu, (2015) | Y | Y | Y | N | Y | Y | Y | Y | Y | Y | Y | Y | Y | N | N | N | Y |
| Man, (2014) | Y | Y | Y | N | Y | Y | Y | Y | Y | N | Y | Y | N | N | N | N | Y |
| Tian and Wang, (2012) | Y | Y | N | Y | Y | Y | Y | Y | Y | Y | Y | Y | N | N | N | N | Y |
| Shi, (2009) | Y | Y | N | Y | Y | N | Y | Y | Y | Y | Y | Y | N | N | N | N | Y |
| Cao and Sun, (2009) | Y | Y | N | N | Ya | Y | N | Y | N | N | Y | Y | N | N | N | N | Y |
| Liu et al., (2008) | Y | Y | N | Y | Y | Y | Y | Y | Y | Y | Y | Y | N | N | N | Y | Y |
| Zhang et al., (2008) | Y | Y | N | N | Ya | Y | Y | Y | Y | Y | Y | Y | N | N | N | N | Y |
| Wang et al., (2008) | Y | Y | N | N | Y | N | Y | Y | Y | Y | Y | Y | N | N | N | N | Y |
| Li and Song, (2005) | Y | Y | N | N | Ya | Y | Y | Y | Y | Y | Y | Y | Y | N | N | N | Y |
| Zhou and Wang, (2001) | Y | Y | N | Y | Y | Y | Y | Y | Y | Y | Y | Y | N | N | N | Y | Y |
| Liu and Wang, (2022) | Y | Y | N | N | Ya | N | N | Y | Y | Y | Y | Y | N | N | N | N | Y |
| Tian, (2022) | Y | Y | N | N | Ya | Y | Y | Y | Y | Y | Y | Y | N | N | N | Y | Y |
| Yang, (2018) | Y | Y | N | N | Ya | Y | Y | N | Y | Y | Y | Y | N | N | N | Y | Y |
| Li et al., (2018) | Y | Y | N | N | Ya | Y | Y | N | Y | Y | Y | Y | N | N | N | Y | Y |
| Xie et al., (2016) | Y | Y | N | Y | Y | N | N | Y | N | N | Y | Y | Y | N | N | Y | Y |
| Zhang et al., (2015) | Y | Y | N | N | Ya | Y | N | Y | N | N | Y | Y | N | N | N | Y | Y |
| Yuan et al., (2014) | Y | Y | N | Y | Y | Y | Y | Y | Y | N | Y | Y | N | N | N | Y | Y |

Note:

1a) Style of acupuncture (e.g. Traditional Chinese Medicine, Japanese, Korean, Western medical, etc);

1b) Reasoning for treatment provided, literature sources, and/or consensus methods, with references where appropriate;

1c) Extent to which treatment was varied;

2a) Number of needle insertions per subject per session (mean and range where relevant) ;

2b) Names (or location if no standard name) of points used (uni/bilateral) ;

2c) Depth of insertion, based on a specified unit of measurement;

2d) Response sought (e.g. de qi or muscle twitch response);

2e) Needle stimulation (e.g. manual，electrical) ;

2f) Needle retention time;

2g) Needle type (diameter, length, and manufacturer) ;

3a) Number of treatment sessions;

3b) Frequency and duration of treatment sessions;

4a) Details of other interventions administered to the acupuncture group (e.g. moxibustion, cupping, herbs, exercises) ;

4b) Setting and context of treatment, including instructions to practitioners, and information and explanations to patients;

5) Description of participating acupuncturists (qualification or professional affiliation, other relevant experience) ;

6a) Rationale for the control or comparator in the context of the research question, with sources that justify this choice;

6b) Precise description of the control or comparator. If sham acupuncture or any other type of acupuncture-like control is used, provide details as for Items 1 to 3 above.

N, not adequately reported; Y, adequately reported; Ya, reported but did not mention unilateral or bilateral.

## Supplementary Table 4. Trim-and-fill test of total responder rate in comparison of acupuncture vs. CT.

| **Outcome** | **Effect-size** | **Effect model** | **Before trim-and-fill** | | **After trim-and-fill** | | **Increased research** |
| --- | --- | --- | --- | --- | --- | --- | --- |
|  |  |  | **Pooled estimate** | **95％CI** | **Pooled estimate** | **95％CI** |  |
| Acupuncture vs. CT | *RR* | FE | 0.143 | 0.098 to 0.187 | 1.106 | 1.062 to 1.151 | 6 |
|  |  | RE | 0.156 | 0.099 to 0.213 | 1.112 | 1.042 to 1.186 |  |

Note: CT, conventional treatment; FE, fixed-effects; RE, random-effects; *RR*, risk ratio.

# Supplementary Figures

## Supplementary Figure 1. Reporting details of STRICTA checklist.


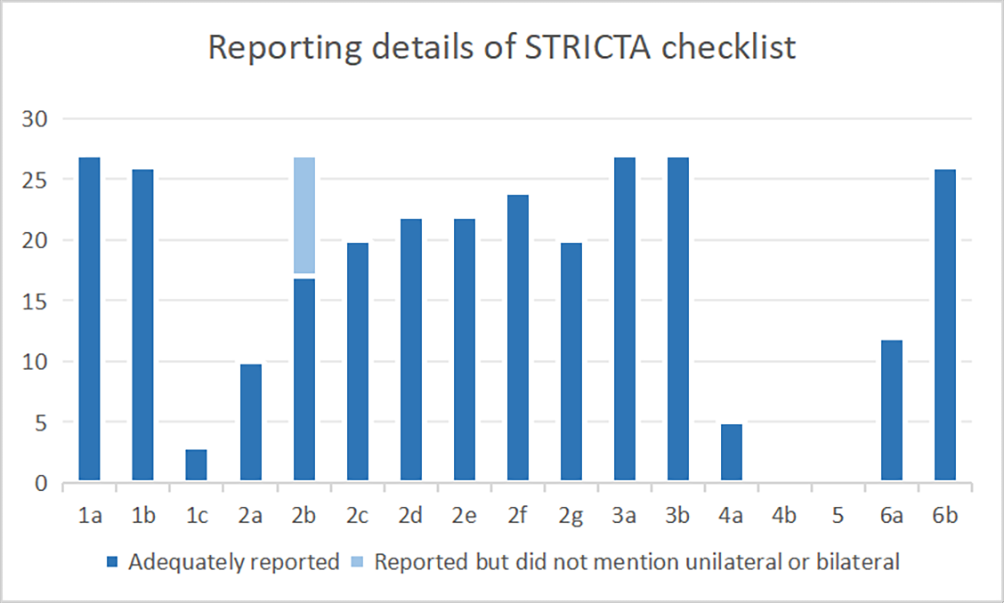


## Supplementary Figure 2. Sensitivity analysis.


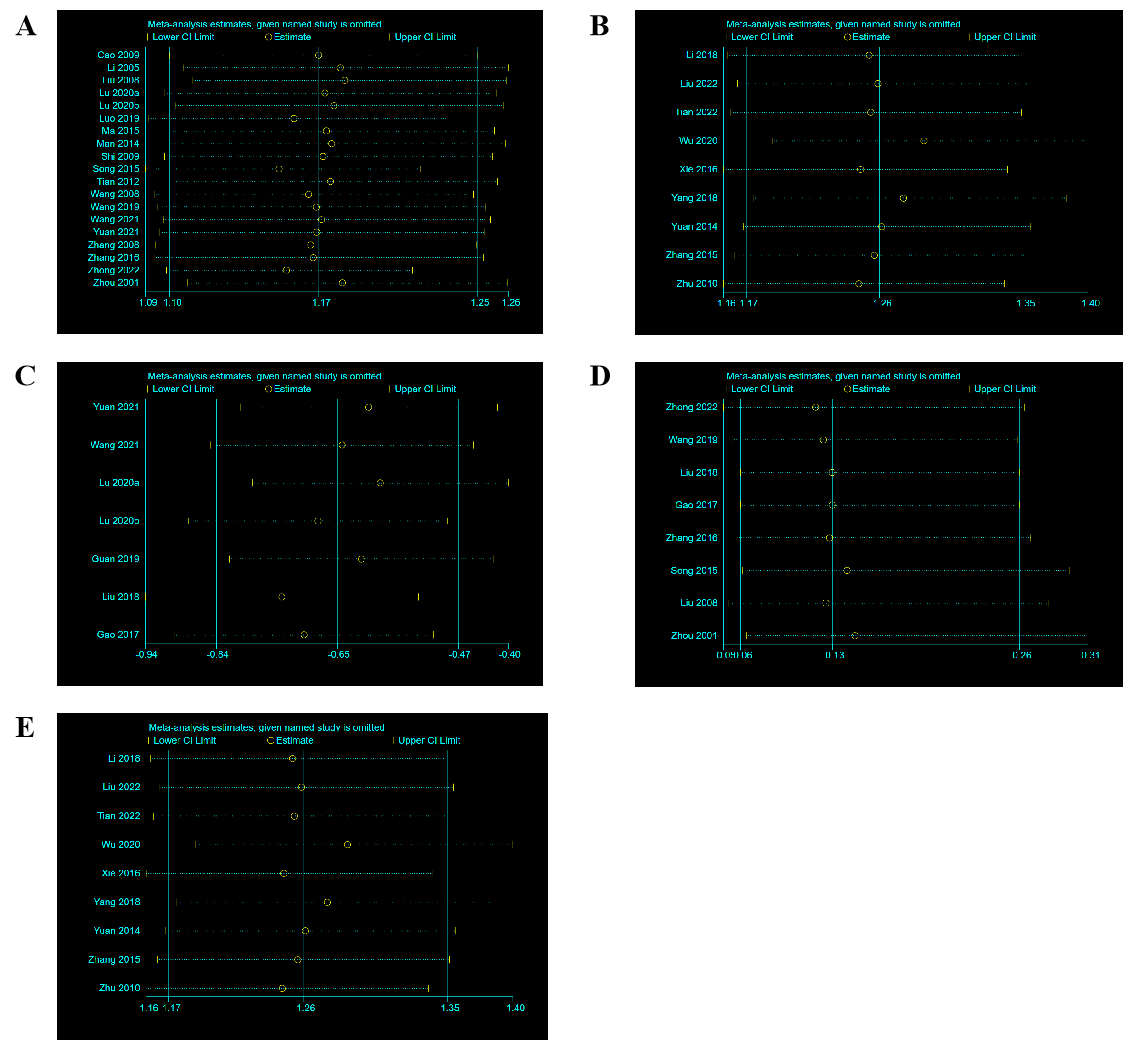


# Supplementary File

## Supplementary File 1. Search strategy.

**CNKI**

((SU=('便秘' + '排便' + '便干' + '大便困难') AND SU=('卒中' + '中风' + '脑出血' + '脑梗死' + '脑血管意外' + '脑梗' + '脑缺血' + '脑梗塞' + '脑血栓' + '脑栓塞' + '缺血性脑卒中' + '缺血性中风' + '蛛网膜下腔出血' + '脑溢血')) OR SU=('卒中后便秘' + '中风后便秘')) AND SU=('针灸' + '针刺' + '温针灸' + '火针' + '电针' + '针' + '刺' + '灸法' + '艾灸' + '灸' + '毫针' + '刺血' + '耳穴' + '耳针' + '头针' + '体针' + '腹针' + '指针' + '穴位' + '中医' + '中西医' + '传统医学' + '结合医学' + '补充替代医学' + '传统医疗' + '替代疗法' + '补充疗法') AND AB=('随机对照试验' + '随机对照研究' + 'RCT' + '随机' + '对照' + '控制组' + '安慰剂' + '试验' + '分组' + '临床' + '临床研究' + '临床疗效')

n = 581

**VIP**

(M=((("便秘" OR "排便" OR "便干" OR "大便困难") AND ("卒中" OR "中风" OR "脑出血" OR "脑梗死" OR "脑血管意外" OR "脑梗" OR "脑缺血" OR "脑梗塞" OR "脑血栓" OR "脑栓塞" OR "缺血性脑卒中" OR "缺血性中风" OR "蛛网膜下腔出血" OR "脑溢血")) OR ("卒中后便秘" OR "中风后便秘")) AND ("针灸" OR "针刺" OR "温针灸" OR "火针" OR "电针" OR "针" OR "刺" OR "灸法" OR "艾灸" OR "灸" OR "刺血" OR "耳穴" OR "耳针" OR "头针" OR "体针" OR "腹针" OR "指针" OR "穴位" OR "中医" OR "中西医" OR "传统医学" OR "结合医学" OR "补充替代医学" OR "传统医疗" OR "替代疗法" OR "补充疗法")) AND ("随机对照试验" OR "随机对照研究" OR "RCT" OR "随机" OR "对照" OR "控制组" OR "安慰剂" OR "试验" OR "分组" OR "临床" OR "临床研究" OR "临床疗效")

n = 191

**Wanfang**

检索表达式： ((主题:("便秘" OR "排便" OR "便干" OR "大便困难") and 主题:("卒中" OR "中风" OR "脑出血" OR "脑梗死" OR "脑血管意外" OR "脑梗" OR "脑缺血" OR "脑梗塞" OR "脑血栓" OR "脑栓塞" OR "缺血性脑卒中" OR "缺血性中风" OR "蛛网膜下腔出血" OR "脑溢血")) or 主题:("卒中后便秘" OR "中风后便秘")) and 主题:("针灸" OR "针刺" OR "温针灸" OR "火针" OR "电针" OR "针" OR "刺" OR "灸法" OR "艾灸" OR "灸" OR "毫针" OR "刺血" OR "耳穴" OR "耳针" OR "头针" OR "体针" OR "腹针" OR "指针" OR "穴位" OR "中医" OR "中西医" OR "传统医学" OR "结合医学" OR "补充替代医学" OR "传统医疗" OR "替代疗法" OR "补充疗法") and 摘要:("随机对照试验" OR "随机对照研究" OR "RCT" OR "随机" OR "对照" OR "控制组" OR "安慰剂" OR "试验" OR "分组" OR "临床" OR "临床研究" OR "临床疗效")

n = 1047

**SinoMed**

(("随机对照试验"[常用字段:智能] OR "随机对照研究"[常用字段:智能] OR "RCT"[常用字段:智能] OR "随机"[常用字段:智能] OR "对照"[常用字段:智能] OR "控制组"[常用字段:智能] OR "安慰剂"[常用字段:智能] OR "试验"[常用字段:智能] OR "分组"[常用字段:智能] OR "临床"[常用字段:智能] OR "临床研究"[常用字段:智能] OR "临床疗效"[常用字段:智能]) OR ("随机对照试验"[不加权:扩展] OR "随机对照试验(主题)"[不加权:扩展])) AND (("针灸"[常用字段:智能] OR "针刺"[常用字段:智能] OR "温针灸"[常用字段:智能] OR "火针"[常用字段:智能] OR "电针"[常用字段:智能] OR "针"[常用字段:智能] OR "刺"[常用字段:智能] OR "灸法"[常用字段:智能] OR "艾灸"[常用字段:智能] OR "灸"[常用字段:智能] OR "刺血"[常用字段:智能] OR "耳穴"[常用字段:智能] OR "耳针"[常用字段:智能] OR "头针"[常用字段:智能] OR "体针"[常用字段:智能] OR "腹针"[常用字段:智能] OR "指针"[常用字段:智能] OR "穴位"[常用字段:智能] OR "中医"[常用字段:智能] OR "中西医"[常用字段:智能] OR "传统医学"[常用字段:智能] OR "结合医学"[常用字段:智能] OR "补充替代医学"[常用字段:智能] OR "传统医疗"[常用字段:智能] OR "替代疗法"[常用字段:智能] OR "补充疗法"[常用字段:智能]) OR ("针灸疗法"[不加权:扩展] OR "温针疗法"[不加权:扩展] OR "逆针灸"[不加权:扩展] OR "太乙针灸疗法"[不加权:扩展] OR "针灸处方"[不加权:扩展] OR "雷火灸疗法"[不加权:扩展] OR "针刺"[不加权:扩展] OR "针刺, 耳"[不加权:扩展] OR "针刺泻法"[不加权:扩展] OR "针刺补法"[不加权:扩展] OR "针刺补泻"[不加权:扩展] OR "排针刺法"[不加权:扩展] OR "放血疗法"[不加权:扩展] OR "微针疗法"[不加权:扩展] OR "火针疗法"[不加权:扩展] OR "电针疗法"[不加权:扩展] OR "电针"[不加权:扩展] OR "灸法"[不加权:扩展] OR "温灸器灸法"[不加权:扩展] OR "头针疗法"[不加权:扩展] OR "体针疗法"[不加权:扩展] OR "腹针疗法"[不加权:扩展] OR "医学, 中国传统"[不加权:扩展] OR "中西医结合"[不加权:扩展] OR "中西医结合疗法"[不加权:扩展] OR "补充疗法"[不加权:扩展] OR "医学, 中国传统"[不加权:扩展] OR "中医学"[不加权:扩展])) AND ((( "卒中后便秘"[常用字段:智能] OR "中风后便秘"[常用字段:智能])) OR (((("脑出血"[常用字段:智能] OR "颅内出血"[常用字段:智能] OR "脑梗死"[常用字段:智能] OR "中风"[常用字段:智能] OR "卒中"[常用字段:智能] OR "缺血性卒中"[常用字段:智能] OR "出血性卒中"[常用字段:智能] OR "栓塞性卒中"[常用字段:智能] OR "颅内栓塞"[常用字段:智能] OR "颅内栓塞和血栓形成"[常用字段:智能] OR "蛛网膜下腔出血"[常用字段:智能] OR "脑梗塞"[常用字段:智能] OR "脑栓塞"[常用字段:智能] OR "脑血栓形成"[常用字段:智能] OR "脑溢血"[常用字段:智能] OR "脑血管意外"[常用字段:智能])) OR (("出血性卒中"[不加权:扩展] OR "栓塞性卒中"[不加权:扩展] OR "颅内栓塞"[不加权:扩展] OR "颅内栓塞和血栓形成"[不加权:扩展] OR "蛛网膜下腔出血"[不加权:扩展]) OR ("脑出血"[不加权:扩展] OR "颅内出血"[不加权:扩展] OR "脑梗死"[不加权:扩展] OR "中风"[不加权:扩展] OR "卒中"[不加权:扩展] OR "缺血性卒中"[不加权:扩展]))) AND ((( "便秘"[常用字段:智能] OR "排便"[常用字段:智能] OR "便干"[常用字段:智能] OR "大便困难"[常用字段:智能])) OR (("便秘"[不加权:扩展] OR "便秘"[不加权:扩展]) OR "排便异常"[不加权:扩展]))))

1032

2023-05-09 23:31:49.0

n = 1032

**PubMed**

((((("Constipation"[Mesh]) OR (Constipation[Title/Abstract] OR Dyschezia[Title/Abstract] OR Colonic Inertia[Title/Abstract] OR Astriction[Title/Abstract])) AND (("Stroke"[Mesh] OR "Cerebral Infarction"[Mesh] OR "Cerebral Hemorrhage"[Mesh] OR "Brain Ischemia"[Mesh] OR "Brain Infarction"[Mesh] OR "Cerebrovascular Disorders"[Mesh] OR "Hemorrhagic Stroke"[Mesh] OR "Ischemic Stroke"[Mesh]) OR ("Stroke"[Title/Abstract] OR "Strokes"[Title/Abstract] OR "Cerebrovascular Accident"[Title/Abstract] OR "Cerebrovascular Accidents"[Title/Abstract] OR "CVA (Cerebrovascular Accident)"[Title/Abstract] OR "CVAs (Cerebrovascular Accident)"[Title/Abstract] OR "Cerebrovascular Apoplexy"[Title/Abstract] OR "Apoplexy, Cerebrovascular"[Title/Abstract] OR "Vascular Accident, Brain"[Title/Abstract] OR "Brain Vascular Accident"[Title/Abstract] OR "Brain Vascular Accidents"[Title/Abstract] OR "Vascular Accidents, Brain"[Title/Abstract] OR "Cerebrovascular Stroke"[Title/Abstract] OR "Cerebrovascular Strokes"[Title/Abstract] OR "Stroke, Cerebrovascular"[Title/Abstract] OR "Strokes, Cerebrovascular"[Title/Abstract] OR "Apoplexy"[Title/Abstract] OR "Cerebral Stroke"[Title/Abstract] OR "Cerebral Strokes"[Title/Abstract] OR "Stroke, Cerebral"[Title/Abstract] OR "Strokes, Cerebral"[Title/Abstract] OR "Stroke, Acute"[Title/Abstract] OR "Acute Stroke"[Title/Abstract] OR "Acute Strokes"[Title/Abstract] OR "Strokes, Acute"[Title/Abstract] OR "Cerebrovascular Accident, Acute"[Title/Abstract] OR "Acute Cerebrovascular Accident"[Title/Abstract] OR "Acute Cerebrovascular Accidents"[Title/Abstract] OR "Cerebrovascular Accidents, Acute"[Title/Abstract] OR "Cerebral Infarction"[Title/Abstract] OR "Cerebral Infarctions"[Title/Abstract] OR "Infarctions, Cerebral"[Title/Abstract] OR "Infarction, Cerebral"[Title/Abstract] OR "Cerebral Infarct"[Title/Abstract] OR "Cerebral Infarcts"[Title/Abstract] OR "Infarct, Cerebral"[Title/Abstract] OR "Infarcts, Cerebral"[Title/Abstract] OR "Cerebral Infarction, Left Hemisphere"[Title/Abstract] OR "Left Hemisphere, Cerebral Infarction"[Title/Abstract] OR "Infarction, Cerebral, Left Hemisphere"[Title/Abstract] OR "Cerebral, Left Hemisphere, Infarction"[Title/Abstract] OR "Infarction, Left Hemisphere, Cerebral"[Title/Abstract] OR "Left Hemisphere, Infarction, Cerebral"[Title/Abstract] OR "Subcortical Infarction"[Title/Abstract] OR "Infarction, Subcortical"[Title/Abstract] OR "Infarctions, Subcortical"[Title/Abstract] OR "Subcortical Infarctions"[Title/Abstract] OR "Posterior Choroidal Artery Infarction"[Title/Abstract] OR "Anterior Choroidal Artery Infarction"[Title/Abstract] OR "Cerebral Infarction, Right Hemisphere"[Title/Abstract] OR "Right Hemisphere, Cerebral Infarction"[Title/Abstract] OR "Infarction, Right Hemisphere, Cerebral"[Title/Abstract] OR "Right Hemisphere, Infarction, Cerebral"[Title/Abstract] OR "Cerebral, Right Hemisphere, Infarction"[Title/Abstract] OR "Infarction, Cerebral, Right Hemisphere"[Title/Abstract] OR "Cerebral Hemorrhage"[Title/Abstract] OR "Hemorrhage, Cerebrum"[Title/Abstract] OR "Cerebrum Hemorrhage"[Title/Abstract] OR "Cerebrum Hemorrhages"[Title/Abstract] OR "Hemorrhages, Cerebrum"[Title/Abstract] OR "Cerebral Parenchymal Hemorrhage"[Title/Abstract] OR "Cerebral Parenchymal Hemorrhages"[Title/Abstract] OR "Hemorrhage, Cerebral Parenchymal"[Title/Abstract] OR "Hemorrhages, Cerebral Parenchymal"[Title/Abstract] OR "Parenchymal Hemorrhage, Cerebral"[Title/Abstract] OR "Parenchymal Hemorrhages, Cerebral"[Title/Abstract] OR "Intracerebral Hemorrhage"[Title/Abstract] OR "Hemorrhage, Intracerebral"[Title/Abstract] OR "Hemorrhages, Intracerebral"[Title/Abstract] OR "Intracerebral Hemorrhages"[Title/Abstract] OR "Hemorrhage, Cerebral"[Title/Abstract] OR "Cerebral Hemorrhages"[Title/Abstract] OR "Hemorrhages, Cerebral"[Title/Abstract] OR "Brain Hemorrhage, Cerebral"[Title/Abstract] OR "Brain Hemorrhages, Cerebral"[Title/Abstract] OR "Cerebral Brain Hemorrhage"[Title/Abstract] OR "Cerebral Brain Hemorrhages"[Title/Abstract] OR "Hemorrhage, Cerebral Brain"[Title/Abstract] OR "Hemorrhages, Cerebral Brain"[Title/Abstract] OR "Hemorrhagic Stroke"[Title/Abstract] OR "Hemorrhagic Strokes"[Title/Abstract] OR "Stroke, Hemorrhagic"[Title/Abstract] OR "Subarachnoid Hemorrhagic Stroke"[Title/Abstract] OR "Hemorrhagic Stroke, Subarachnoid"[Title/Abstract] OR "Stroke, Subarachnoid Hemorrhagic"[Title/Abstract] OR "Subarachnoid Hemorrhagic Strokes"[Title/Abstract] OR "Intracerebral Hemorrhagic Stroke"[Title/Abstract] OR "Hemorrhagic Stroke, Intracerebral"[Title/Abstract] OR "Intracerebral Hemorrhagic Strokes"[Title/Abstract] OR "Stroke, Intracerebral Hemorrhagic"[Title/Abstract] OR "Intracerebral Hemorrhage Stroke"[Title/Abstract] OR "Hemorrhage Stroke, Intracerebral"[Title/Abstract] OR "Intracerebral Hemorrhage Strokes"[Title/Abstract] OR "Stroke, Intracerebral Hemorrhage"[Title/Abstract] OR "Ischemic Stroke"[Title/Abstract] OR "Ischemic Strokes"[Title/Abstract] OR "Stroke, Ischemic"[Title/Abstract] OR "Ischaemic Stroke"[Title/Abstract] OR "Ischaemic Strokes"[Title/Abstract] OR "Stroke, Ischaemic"[Title/Abstract] OR "Cryptogenic Ischemic Stroke"[Title/Abstract] OR "Cryptogenic Ischemic Strokes"[Title/Abstract] OR "Ischemic Stroke, Cryptogenic"[Title/Abstract] OR "Stroke, Cryptogenic Ischemic"[Title/Abstract] OR "Cryptogenic Stroke"[Title/Abstract] OR "Cryptogenic Strokes"[Title/Abstract] OR "Stroke, Cryptogenic"[Title/Abstract] OR "Cryptogenic Embolism Stroke"[Title/Abstract] OR "Cryptogenic Embolism Strokes"[Title/Abstract] OR "Embolism Stroke, Cryptogenic"[Title/Abstract] OR "Stroke, Cryptogenic Embolism"[Title/Abstract] OR "Wake-up Stroke"[Title/Abstract] OR "Stroke, Wake-up"[Title/Abstract] OR "Wake up Stroke"[Title/Abstract] OR "Wake-up Strokes"[Title/Abstract] OR "Acute Ischemic Stroke"[Title/Abstract] OR "Acute Ischemic Strokes"[Title/Abstract] OR "Ischemic Stroke, Acute"[Title/Abstract] OR "Stroke, Acute Ischemic"[Title/Abstract] OR "Brain ischemia"[Title/Abstract] OR "Brain Ischemias"[Title/Abstract] OR "Ischemia, Brain"[Title/Abstract] OR "Ischemic Encephalopathy"[Title/Abstract] OR "Encephalopathy, Ischemic"[Title/Abstract] OR "Ischemic Encephalopathies"[Title/Abstract] OR "Cerebral Ischemia"[Title/Abstract] OR "Cerebral Ischemias"[Title/Abstract] OR "Ischemias, Cerebral"[Title/Abstract] OR "Ischemia, Cerebral"[Title/Abstract] OR "Brain Infarction"[Title/Abstract] OR "Brain Infarctions"[Title/Abstract] OR "Infarction, Brain"[Title/Abstract] OR "Infarctions, Brain"[Title/Abstract] OR "Brain Infarct"[Title/Abstract] OR "Brain Infarcts"[Title/Abstract] OR "Infarct, Brain"[Title/Abstract] OR "Infarcts, Brain"[Title/Abstract] OR "Brain Infarction, Posterior Circulation"[Title/Abstract] OR "Infarction, Posterior Circulation, Brain"[Title/Abstract] OR "Posterior Circulation Infarction, Brain"[Title/Abstract] OR "Infarction, Brain, Posterior Circulation"[Title/Abstract] OR "Posterior Circulation Brain Infarction"[Title/Abstract] OR "Anterior Circulation Brain Infarction"[Title/Abstract] OR "Infarction, Brain, Anterior Circulation"[Title/Abstract] OR "Brain Infarction, Anterior Circulation"[Title/Abstract] OR "Anterior Circulation Infarction, Brain"[Title/Abstract] OR "Infarction, Anterior Circulation, Brain"[Title/Abstract] OR "Venous Infarction, Brain"[Title/Abstract] OR "Brain Venous Infarction"[Title/Abstract] OR "Brain Venous Infarctions"[Title/Abstract] OR "Infarction, Brain Venous"[Title/Abstract] OR "Infarctions, Brain Venous"[Title/Abstract] OR "Venous Infarctions, Brain"[Title/Abstract] OR "Brain Infarction, Venous"[Title/Abstract] OR "Brain Infarctions, Venous"[Title/Abstract] OR "Infarction, Venous Brain"[Title/Abstract] OR "Infarctions, Venous Brain"[Title/Abstract] OR "Venous Brain Infarction"[Title/Abstract] OR "Venous Brain Infarctions"[Title/Abstract] OR "Anterior Cerebral Circulation Infarction"[Title/Abstract] OR "Infarction, Anterior Cerebral Circulation"[Title/Abstract] OR "Cerebrovascular Disorders"[Title/Abstract] OR "Cerebrovascular Disorder"[Title/Abstract] OR "Cerebrovascular Diseases"[Title/Abstract] OR "Cerebrovascular Disease"[Title/Abstract] OR "Disease, Cerebrovascular"[Title/Abstract] OR "Diseases, Cerebrovascular"[Title/Abstract] OR "Vascular Diseases, Intracranial"[Title/Abstract] OR "Intracranial Vascular Disease"[Title/Abstract] OR "Intracranial Vascular Diseases"[Title/Abstract] OR "Vascular Disease, Intracranial"[Title/Abstract] OR "Brain Vascular Disorders"[Title/Abstract] OR "Brain Vascular Disorder"[Title/Abstract] OR "Vascular Disorder, Brain"[Title/Abstract] OR "Vascular Disorders, Brain"[Title/Abstract] OR "Intracranial Vascular Disorders"[Title/Abstract] OR "Intracranial Vascular Disorder"[Title/Abstract] OR "Vascular Disorder, Intracranial"[Title/Abstract] OR "Vascular Disorders, Intracranial"[Title/Abstract] OR "Cerebrovascular Insufficiency"[Title/Abstract] OR "Cerebrovascular Insufficiencies"[Title/Abstract] OR "Insufficiencies, Cerebrovascular"[Title/Abstract] OR "Insufficiency, Cerebrovascular"[Title/Abstract] OR "Cerebrovascular Occlusion"[Title/Abstract] OR "Cerebrovascular Occlusions"[Title/Abstract] OR "Occlusion, Cerebrovascular"[Title/Abstract] OR "Occlusions, Cerebrovascular"[Title/Abstract] OR "CVA"[Title/Abstract] OR "Intracranial Thromb*"[Title/Abstract] OR "Ischemic Event"[Title/Abstract] OR "Brain Thrombosis"[Title/Abstract] OR "Cerebral Thrombus"[Title/Abstract] OR "Ischemic Encephalopathies"[Title/Abstract] OR "Ischemic Attack"[Title/Abstract]))) OR ("Post stroke constipation"[Title/Abstract] OR "Poststroke constipation"[Title/Abstract])) AND (((((((("Acupuncture"[Mesh] OR "Acupuncture Therapy"[Mesh] OR "Acupuncture, Ear"[Mesh] OR "Acupuncture Points"[Mesh]) OR "Auriculotherapy"[Mesh]) OR "Electroacupuncture"[Mesh]) OR "Dry Needling"[Mesh]) OR "Moxibustion"[Mesh]) OR "Meridians"[Mesh]) OR "Complementary Therapies"[Mesh]) OR ("Acupuncture"[Title/Abstract] OR "Acupuncture Therapy"[Title/Abstract] OR "Acupuncture Treatment*"[Title/Abstract] OR "Treatment, Acupuncture"[Title/Abstract] OR "Therapy, Acupuncture"[Title/Abstract] OR "Pharmacoacupuncture Treatment"[Title/Abstract] OR "Treatment, Pharmacoacupuncture"[Title/Abstract] OR "Pharmacoacupuncture Therapy"[Title/Abstract] OR "Therapy, Pharmacoacupuncture"[Title/Abstract] OR "Acupotom*"[Title/Abstract] OR "Acupuncture*, Ear"[Title/Abstract] OR "Ear Acupuncture*"[Title/Abstract] OR "Auricular Acupuncture*"[Title/Abstract] OR "Acupuncture*, Auricular"[Title/Abstract] OR "Acupuncture Point*"[Title/Abstract] OR "Point*, Acupuncture"[Title/Abstract] OR "Acupoint*"[Title/Abstract] OR "Auriculotherap*"[Title/Abstract] OR "electroacupuncture"[Title/Abstract] OR "Dry Needling"[Title/Abstract] OR "Needling, Dry"[Title/Abstract] OR "moxibustion"[Title/Abstract] OR "Moxabustion"[Title/Abstract] OR "meridians"[Title/Abstract] OR "Ching Lo"[Title/Abstract] OR "Jing Luo"[Title/Abstract] OR "Luo, Jing"[Title/Abstract] OR "Jingluo"[Title/Abstract] OR "Complementary Therap*"[Title/Abstract] OR "Therap*, Complementary"[Title/Abstract] OR "Complementary Medicine"[Title/Abstract] OR "Medicine, Complementary"[Title/Abstract] OR "Alternative Medicine"[Title/Abstract] OR "Medicine, Alternative"[Title/Abstract] OR "Alternative Therap*"[Title/Abstract] OR "Therap*, Alternative"[Title/Abstract] OR "electric-acupuncture"[Title/Abstract] OR "electro-acupuncture"[Title/Abstract] OR "warm acupuncture"[Title/Abstract] OR "scalp acupuncture"[Title/Abstract] OR "Integrat* Therap*"[Title/Abstract]))) AND ((("Randomized Controlled Trial" [Publication Type] OR "Randomized Controlled Trials as Topic"[Mesh]) OR "Controlled Clinical Trial" [Publication Type]) OR ((((((((((((((((((("Randomized Controlled Trial"[Title/Abstract]) OR ("Randomized Controlled Trials as Topic"[Title/Abstract])) OR ("Controlled Clinical Trial"[Title/Abstract])) OR ("Randomized"[Title/Abstract])) OR ("placebo"[Title/Abstract])) OR ("randomly"[Title/Abstract])) OR ("trial"[Title/Abstract])) OR ("groups"[Title/Abstract])) OR ("Clinical Trials"[Title/Abstract])) OR ("Random"[Title/Abstract])) OR ("RCT"[Title/Abstract])) OR ("randomized controlled study"[Title/Abstract])) OR ("Controlled Clinical study"[Title/Abstract])) OR ("randomized trial"[Title/Abstract])) OR ("randomized study"[Title/Abstract])) OR ("randomized placebo-controlled study"[Title/Abstract])) OR ("randomized parallel-group study"[Title/Abstract])) OR ("randomized placebo controlled"[Title/Abstract])) OR ("randomized double-blin*"[Title/Abstract])))

n = 25

**Embase**

SourcesEmbase, MEDLINE, Preprints

Query(('constipation'/exp OR 'constipation':ab,ti,kw OR 'dyschezia':ab,ti,kw OR 'colonic inertia':ab,ti,kw OR 'astriction':ab,ti,kw) AND ('cerebrovascular accident'/exp OR 'brain infarction'/exp OR 'brain hemorrhage'/exp OR 'brain ischemia'/exp OR 'ischemic stroke'/exp OR 'occlusive cerebrovascular disease'/exp OR 'cerebrovascular accident':ab,ti,kw OR 'brainstem stroke':ab,ti,kw OR 'cardioembolic stroke':ab,ti,kw OR 'experimental stroke':ab,ti,kw OR 'lacunar stroke':ab,ti,kw OR 'brain infarction':ab,ti,kw OR 'anterior circulation infarction':ab,ti,kw OR 'brain infarction size':ab,ti,kw OR 'brain stem infarction':ab,ti,kw OR 'cadasil':ab,ti,kw OR 'cerebellum infarction':ab,ti,kw OR 'lacunar infarction':ab,ti,kw OR 'migrainous infarction':ab,ti,kw OR 'multiinfarct dementia':ab,ti,kw OR 'posterior circulation infarction':ab,ti,kw OR 'brain hemorrhage':ab,ti,kw OR 'brain ventricle hemorrhage':ab,ti,kw OR 'cerebellum hemorrhage':ab,ti,kw OR 'massive intracerebral hemorrhage':ab,ti,kw OR 'subarachnoid hemorrhage':ab,ti,kw OR 'brain ischemia':ab,ti,kw OR 'anterior circulation ischemia':ab,ti,kw OR 'brain vasospasm':ab,ti,kw OR 'experimental cerebral ischemia':ab,ti,kw OR 'hypoxic ischemic encephalopathy':ab,ti,kw OR 'posterior circulation ischemia':ab,ti,kw OR 'transient ischemic attack':ab,ti,kw OR 'ischemic stroke':ab,ti,kw OR 'acute ischemic stroke':ab,ti,kw OR 'anterior circulation stroke':ab,ti,kw OR 'chronic ischemic stroke':ab,ti,kw OR 'cryptogenic ischemic stroke':ab,ti,kw OR 'posterior circulation stroke':ab,ti,kw OR 'subacute ischemic stroke':ab,ti,kw OR 'wake up stroke':ab,ti,kw OR 'occlusive cerebrovascular disease':ab,ti,kw OR 'basilar artery obstruction':ab,ti,kw OR 'cerebral sinus thrombosis':ab,ti,kw OR 'middle cerebral artery occlusion':ab,ti,kw OR 'sneddon syndrome':ab,ti,kw OR 'susac syndrome':ab,ti,kw OR 'vertebral artery stenosis':ab,ti,kw OR 'intracranial thromb*':ab,ti,kw OR 'ischemic event':ab,ti,kw OR 'brain thrombosis':ab,ti,kw OR 'cerebral thrombus':ab,ti,kw OR 'ischemic encephalopathies':ab,ti,kw OR 'ischemic attack':ab,ti,kw) OR 'poststroke constipation':ab,ti,kw OR 'post-stroke constipation':ab,ti,kw) AND ('acupuncture'/exp OR 'auricular acupuncture'/exp OR 'acupuncture point'/exp OR 'electroacupuncture'/exp OR 'dry needling'/exp OR 'moxibustion'/exp OR 'body meridian'/exp OR 'alternative medicine'/exp OR 'warm acupuncture'/exp OR 'acupuncture':ab,ti,kw OR 'acupressure':ab,ti,kw OR 'acupuncture analgesia':ab,ti,kw OR 'catgut embedding':ab,ti,kw OR 'pharmacopuncture':ab,ti,kw OR 'auricular acupuncture':ab,ti,kw OR 'bladder meridian acupoint':ab,ti,kw OR 'conception vessel acupoint':ab,ti,kw OR 'gallbladder meridian acupoint':ab,ti,kw OR 'governor vessel acupoint':ab,ti,kw OR 'heart meridian acupoint':ab,ti,kw OR 'kidney meridian acupoint':ab,ti,kw OR 'large intestine meridian acupoint':ab,ti,kw OR 'liver meridian acupoint':ab,ti,kw OR 'lung meridian acupoint':ab,ti,kw OR 'pericardium meridian acupoint':ab,ti,kw OR 'small intestine meridian acupoint':ab,ti,kw OR 'spleen meridian acupoint':ab,ti,kw OR 'stomach meridian acupoint':ab,ti,kw OR 'triple energizer meridian acupoint':ab,ti,kw OR 'electroacupuncture':ab,ti,kw OR 'dry needling':ab,ti,kw OR 'moxibustion':ab,ti,kw OR 'body meridian':ab,ti,kw OR 'extraordinary meridian':ab,ti,kw OR 'principal meridian (chinese medicine)':ab,ti,kw OR 'alternative medicine':ab,ti,kw OR 'warm acupuncture':ab,ti,kw OR 'electric-acupuncture':ab,ti,kw OR 'electro-acupuncture':ab,ti,kw OR 'scalp acupuncture':ab,ti,kw OR 'integrat* therap*':ab,ti,kw) AND ('randomized controlled trial'/exp OR 'randomized controlled trial (topic)'/exp OR 'controlled clinical trial'/exp OR 'randomized controlled trial':ab,ti,kw OR 'randomized controlled trial (topic)':ab,ti,kw OR 'controlled clinical trial':ab,ti,kw OR 'randomized':ab,ti,kw OR 'placebo':ab,ti,kw OR 'randomly':ab,ti,kw OR 'trial':ab,ti,kw OR 'groups':ab,ti,kw OR 'clinical trials':ab,ti,kw OR 'random':ab,ti,kw OR 'rct':ab,ti,kw OR 'randomized controlled study':ab,ti,kw OR 'randomized trial':ab,ti,kw OR 'randomized study':ab,ti,kw OR 'randomized placebo-controlled study':ab,ti,kw OR 'randomized parallel-group study':ab,ti,kw OR 'randomized placebo controlled':ab,ti,kw OR 'randomized double-blind':ab,ti,kw)

Mapped termsn/a

n = 44

**Cochrane Library**

Search Name: Poststroke constipation

Date Run: 09/05/2023 23:09:34

Comment:

ID Search Hits

#1 MeSH descriptor: [Constipation] explode all trees 3430

#2 ("constipation"):ti,ab,kw OR ("Dyschezia"):ti,ab,kw OR ("Colonic Inertia"):ti,ab,kw OR ("Astriction"):ti,ab,kw 14883

#3 #1 OR #2 14883

#4 MeSH descriptor: [Stroke] explode all trees 14721

#5 MeSH descriptor: [Cerebral Infarction] explode all trees 1467

#6 MeSH descriptor: [Cerebral Hemorrhage] explode all trees 1365

#7 MeSH descriptor: [Brain Ischemia] explode all trees 5899

#8 MeSH descriptor: [Brain Infarction] explode all trees 1905

#9 MeSH descriptor: [Cerebrovascular Disorders] explode all trees 22365

#10 MeSH descriptor: [Hemorrhagic Stroke] explode all trees 34

#11 MeSH descriptor: [Ischemic Stroke] explode all trees 862

#12 #4 OR #5 OR #6 OR #7 OR #8 OR #9 OR #10 OR #11 22365

#13 ("Stroke"):ti,ab,kw OR ("Strokes"):ti,ab,kw OR ("Cerebrovascular Accident"):ti,ab,kw OR ("Cerebrovascular Accidents"):ti,ab,kw OR ("CVA (Cerebrovascular Accident)"):ti,ab,kw OR ("CVAs (Cerebrovascular Accident)"):ti,ab,kw OR ("Cerebrovascular Apoplexy"):ti,ab,kw OR ("Apoplexy, Cerebrovascular"):ti,ab,kw OR ("Vascular Accident, Brain"):ti,ab,kw OR ("Brain Vascular Accident"):ti,ab,kw OR ("Brain Vascular Accidents"):ti,ab,kw OR ("Vascular Accidents, Brain"):ti,ab,kw OR ("Cerebrovascular Stroke"):ti,ab,kw OR ("Cerebrovascular Strokes"):ti,ab,kw OR ("Stroke, Cerebrovascular"):ti,ab,kw OR ("Strokes, Cerebrovascular"):ti,ab,kw OR ("Apoplexy"):ti,ab,kw OR ("Cerebral Stroke"):ti,ab,kw OR ("Cerebral Strokes"):ti,ab,kw OR ("Stroke, Cerebral"):ti,ab,kw OR ("Strokes, Cerebral"):ti,ab,kw OR ("Stroke, Acute"):ti,ab,kw OR ("Acute Stroke"):ti,ab,kw OR ("Acute Strokes"):ti,ab,kw OR ("Strokes, Acute"):ti,ab,kw OR ("Cerebrovascular Accident, Acute"):ti,ab,kw OR ("Acute Cerebrovascular Accident"):ti,ab,kw OR ("Acute Cerebrovascular Accidents"):ti,ab,kw OR ("Cerebrovascular Accidents, Acute"):ti,ab,kw OR ("Cerebral Infarction"):ti,ab,kw OR ("Cerebral Infarctions"):ti,ab,kw OR ("Infarctions, Cerebral"):ti,ab,kw OR ("Infarction, Cerebral"):ti,ab,kw OR ("Cerebral Infarct"):ti,ab,kw OR ("Cerebral Infarcts"):ti,ab,kw OR ("Infarct, Cerebral"):ti,ab,kw OR ("Infarcts, Cerebral"):ti,ab,kw OR ("Cerebral Infarction, Left Hemisphere"):ti,ab,kw OR ("Left Hemisphere, Cerebral Infarction"):ti,ab,kw OR ("Infarction, Cerebral, Left Hemisphere"):ti,ab,kw OR ("Cerebral, Left Hemisphere, Infarction"):ti,ab,kw OR ("Infarction, Left Hemisphere, Cerebral"):ti,ab,kw OR ("Left Hemisphere, Infarction, Cerebral"):ti,ab,kw OR ("Subcortical Infarction"):ti,ab,kw OR ("Infarction, Subcortical"):ti,ab,kw OR ("Infarctions, Subcortical"):ti,ab,kw OR ("Subcortical Infarctions"):ti,ab,kw OR ("Posterior Choroidal Artery Infarction"):ti,ab,kw OR ("Anterior Choroidal Artery Infarction"):ti,ab,kw OR ("Cerebral Infarction, Right Hemisphere"):ti,ab,kw OR ("Right Hemisphere, Cerebral Infarction"):ti,ab,kw OR ("Infarction, Right Hemisphere, Cerebral"):ti,ab,kw OR ("Right Hemisphere, Infarction, Cerebral"):ti,ab,kw OR ("Cerebral, Right Hemisphere, Infarction"):ti,ab,kw OR ("Infarction, Cerebral, Right Hemisphere"):ti,ab,kw OR ("Cerebral Hemorrhage"):ti,ab,kw OR ("Hemorrhage, Cerebrum"):ti,ab,kw OR ("Cerebrum Hemorrhage"):ti,ab,kw OR ("Cerebrum Hemorrhages"):ti,ab,kw OR ("Hemorrhages, Cerebrum"):ti,ab,kw OR ("Cerebral Parenchymal Hemorrhage"):ti,ab,kw OR ("Cerebral Parenchymal Hemorrhages"):ti,ab,kw OR ("Hemorrhage, Cerebral Parenchymal"):ti,ab,kw OR ("Hemorrhages, Cerebral Parenchymal"):ti,ab,kw OR ("Parenchymal Hemorrhage, Cerebral"):ti,ab,kw OR ("Parenchymal Hemorrhages, Cerebral"):ti,ab,kw OR ("Intracerebral Hemorrhage"):ti,ab,kw OR ("Hemorrhage, Intracerebral"):ti,ab,kw OR ("Hemorrhages, Intracerebral"):ti,ab,kw OR ("Intracerebral Hemorrhages"):ti,ab,kw OR ("Hemorrhage, Cerebral"):ti,ab,kw OR ("Cerebral Hemorrhages"):ti,ab,kw OR ("Hemorrhages, Cerebral"):ti,ab,kw OR ("Brain Hemorrhage, Cerebral"):ti,ab,kw OR ("Brain Hemorrhages, Cerebral"):ti,ab,kw OR ("Cerebral Brain Hemorrhage"):ti,ab,kw OR ("Cerebral Brain Hemorrhages"):ti,ab,kw OR ("Hemorrhage, Cerebral Brain"):ti,ab,kw OR ("Hemorrhages, Cerebral Brain"):ti,ab,kw OR ("Hemorrhagic Stroke"):ti,ab,kw OR ("Hemorrhagic Strokes"):ti,ab,kw OR ("Stroke, Hemorrhagic"):ti,ab,kw OR ("Subarachnoid Hemorrhagic Stroke"):ti,ab,kw OR ("Hemorrhagic Stroke, Subarachnoid"):ti,ab,kw OR ("Stroke, Subarachnoid Hemorrhagic"):ti,ab,kw OR ("Subarachnoid Hemorrhagic Strokes"):ti,ab,kw OR ("Intracerebral Hemorrhagic Stroke"):ti,ab,kw OR ("Hemorrhagic Stroke, Intracerebral"):ti,ab,kw OR ("Intracerebral Hemorrhagic Strokes"):ti,ab,kw OR ("Stroke, Intracerebral Hemorrhagic"):ti,ab,kw OR ("Intracerebral Hemorrhage Stroke"):ti,ab,kw OR ("Hemorrhage Stroke, Intracerebral"):ti,ab,kw OR ("Intracerebral Hemorrhage Strokes"):ti,ab,kw OR ("Stroke, Intracerebral Hemorrhage"):ti,ab,kw OR ("Ischemic Stroke"):ti,ab,kw OR ("Ischemic Strokes"):ti,ab,kw OR ("Stroke, Ischemic"):ti,ab,kw OR ("Ischaemic Stroke"):ti,ab,kw OR ("Ischaemic Strokes"):ti,ab,kw OR ("Stroke, Ischaemic"):ti,ab,kw OR ("Cryptogenic Ischemic Stroke"):ti,ab,kw OR ("Cryptogenic Ischemic Strokes"):ti,ab,kw OR ("Ischemic Stroke, Cryptogenic"):ti,ab,kw OR ("Stroke, Cryptogenic Ischemic"):ti,ab,kw OR ("Cryptogenic Stroke"):ti,ab,kw OR ("Cryptogenic Strokes"):ti,ab,kw OR ("Stroke, Cryptogenic"):ti,ab,kw OR ("Cryptogenic Embolism Stroke"):ti,ab,kw OR ("Cryptogenic Embolism Strokes"):ti,ab,kw OR ("Embolism Stroke, Cryptogenic"):ti,ab,kw OR ("Stroke, Cryptogenic Embolism"):ti,ab,kw OR ("Wake-up Stroke"):ti,ab,kw OR ("Stroke, Wake-up"):ti,ab,kw OR ("Wake up Stroke"):ti,ab,kw OR ("Wake-up Strokes"):ti,ab,kw OR ("Acute Ischemic Stroke"):ti,ab,kw OR ("Acute Ischemic Strokes"):ti,ab,kw OR ("Ischemic Stroke, Acute"):ti,ab,kw OR ("Stroke, Acute Ischemic"):ti,ab,kw OR ("Brain ischemia"):ti,ab,kw OR ("Brain Ischemias"):ti,ab,kw OR ("Ischemia, Brain"):ti,ab,kw OR ("Ischemic Encephalopathy"):ti,ab,kw OR ("Encephalopathy, Ischemic"):ti,ab,kw OR ("Ischemic Encephalopathies"):ti,ab,kw OR ("Cerebral Ischemia"):ti,ab,kw OR ("Cerebral Ischemias"):ti,ab,kw OR ("Ischemias, Cerebral"):ti,ab,kw OR ("Ischemia, Cerebral"):ti,ab,kw OR ("Brain Infarction"):ti,ab,kw OR ("Brain Infarctions"):ti,ab,kw OR ("Infarction, Brain"):ti,ab,kw OR ("Infarctions, Brain"):ti,ab,kw OR ("Brain Infarct"):ti,ab,kw OR ("Brain Infarcts"):ti,ab,kw OR ("Infarct, Brain"):ti,ab,kw OR ("Infarcts, Brain"):ti,ab,kw OR ("Brain Infarction, Posterior Circulation"):ti,ab,kw OR ("Infarction, Posterior Circulation, Brain"):ti,ab,kw OR ("Posterior Circulation Infarction, Brain"):ti,ab,kw OR ("Infarction, Brain, Posterior Circulation"):ti,ab,kw OR ("Posterior Circulation Brain Infarction"):ti,ab,kw OR ("Anterior Circulation Brain Infarction"):ti,ab,kw OR ("Infarction, Brain, Anterior Circulation"):ti,ab,kw OR ("Brain Infarction, Anterior Circulation"):ti,ab,kw OR ("Anterior Circulation Infarction, Brain"):ti,ab,kw OR ("Infarction, Anterior Circulation, Brain"):ti,ab,kw OR ("Venous Infarction, Brain"):ti,ab,kw OR ("Brain Venous Infarction"):ti,ab,kw OR ("Brain Venous Infarctions"):ti,ab,kw OR ("Infarction, Brain Venous"):ti,ab,kw OR ("Infarctions, Brain Venous"):ti,ab,kw OR ("Venous Infarctions, Brain"):ti,ab,kw OR ("Brain Infarction, Venous"):ti,ab,kw OR ("Brain Infarctions, Venous"):ti,ab,kw OR ("Infarction, Venous Brain"):ti,ab,kw OR ("Infarctions, Venous Brain"):ti,ab,kw OR ("Venous Brain Infarction"):ti,ab,kw OR ("Venous Brain Infarctions"):ti,ab,kw OR ("Anterior Cerebral Circulation Infarction"):ti,ab,kw OR ("Infarction, Anterior Cerebral Circulation"):ti,ab,kw OR ("Cerebrovascular Disorders"):ti,ab,kw OR ("Cerebrovascular Disorder"):ti,ab,kw OR ("Cerebrovascular Diseases"):ti,ab,kw OR ("Cerebrovascular Disease"):ti,ab,kw OR ("Disease, Cerebrovascular"):ti,ab,kw OR ("Diseases, Cerebrovascular"):ti,ab,kw OR ("Vascular Diseases, Intracranial"):ti,ab,kw OR ("Intracranial Vascular Disease"):ti,ab,kw OR ("Intracranial Vascular Diseases"):ti,ab,kw OR ("Vascular Disease, Intracranial"):ti,ab,kw OR ("Brain Vascular Disorders"):ti,ab,kw OR ("Brain Vascular Disorder"):ti,ab,kw OR ("Vascular Disorder, Brain"):ti,ab,kw OR ("Vascular Disorders, Brain"):ti,ab,kw OR ("Intracranial Vascular Disorders"):ti,ab,kw OR ("Intracranial Vascular Disorder"):ti,ab,kw OR ("Vascular Disorder, Intracranial"):ti,ab,kw OR ("Vascular Disorders, Intracranial"):ti,ab,kw OR ("Cerebrovascular Insufficiency"):ti,ab,kw OR ("Cerebrovascular Insufficiencies"):ti,ab,kw OR ("Insufficiencies, Cerebrovascular"):ti,ab,kw OR ("Insufficiency, Cerebrovascular"):ti,ab,kw OR ("Cerebrovascular Occlusion"):ti,ab,kw OR ("Cerebrovascular Occlusions"):ti,ab,kw OR ("Occlusion, Cerebrovascular"):ti,ab,kw OR ("Occlusions, Cerebrovascular"):ti,ab,kw OR ("CVA"):ti,ab,kw OR ("Intracranial Thromb*"):ti,ab,kw OR ("Ischemic Event "):ti,ab,kw OR ("Brain Thrombosis"):ti,ab,kw OR ("Cerebral Thrombus"):ti,ab,kw OR ("Ischemic Encephalopathies"):ti,ab,kw OR ("Ischemic Attack"):ti,ab,kw 80801

#14 #12 OR #13 83513

#15 #3 AND #14 410

#16 ("Post stroke constipation"):ti,ab,kw OR ("Poststroke constipation"):ti,ab,kw 7

#17 #15 OR #16 410

#18 MeSH descriptor: [Acupuncture] explode all trees 713

#19 MeSH descriptor: [Acupuncture Therapy] explode all trees 6380

#20 MeSH descriptor: [Acupuncture, Ear] explode all trees 240

#21 MeSH descriptor: [Acupuncture Points] explode all trees 2494

#22 MeSH descriptor: [Auriculotherapy] explode all trees 282

#23 MeSH descriptor: [Electroacupuncture] explode all trees 1150

#24 MeSH descriptor: [Dry Needling] explode all trees 158

#25 MeSH descriptor: [Moxibustion] explode all trees 656

#26 MeSH descriptor: [Meridians] explode all trees 2549

#27 MeSH descriptor: [Complementary Therapies] explode all trees 26849

#28 #18 OR #19 OR #20 OR #21 OR #22 OR #23 OR #24 OR #25 OR #26 OR #27 27354

#29 ("Acupuncture"):ti,ab,kw OR ("Acupuncture Therapy"):ti,ab,kw OR ("Acupuncture Treatment*"):ti,ab,kw OR ("Treatment, Acupuncture"):ti,ab,kw OR ("Therapy, Acupuncture"):ti,ab,kw OR ("Pharmacoacupuncture Treatment"):ti,ab,kw OR ("Treatment, Pharmacoacupuncture"):ti,ab,kw OR ("Pharmacoacupuncture Therapy"):ti,ab,kw OR ("Therapy, Pharmacoacupuncture"):ti,ab,kw OR ("Acupotom*"):ti,ab,kw OR ("Acupuncture*, Ear"):ti,ab,kw OR ("Ear Acupuncture*"):ti,ab,kw OR ("Auricular Acupuncture*"):ti,ab,kw OR ("Acupuncture*, Auricular"):ti,ab,kw OR ("Acupuncture Point*"):ti,ab,kw OR ("Point*, Acupuncture"):ti,ab,kw OR ("Acupoint*"):ti,ab,kw OR ("Auriculotherap*"):ti,ab,kw OR ("electroacupuncture"):ti,ab,kw OR ("Dry Needling"):ti,ab,kw OR ("Needling, Dry"):ti,ab,kw OR ("moxibustion"):ti,ab,kw OR ("Moxabustion"):ti,ab,kw OR ("meridians"):ti,ab,kw OR ("Ching Lo"):ti,ab,kw OR ("Jing Luo"):ti,ab,kw OR ("Luo, Jing"):ti,ab,kw OR ("Jingluo"):ti,ab,kw OR ("Complementary Therap*"):ti,ab,kw OR ("Therap*, Complementary"):ti,ab,kw OR ("Complementary Medicine"):ti,ab,kw OR ("Medicine, Complementary"):ti,ab,kw OR ("Alternative Medicine"):ti,ab,kw OR ("Medicine, Alternative"):ti,ab,kw OR ("Alternative Therap*"):ti,ab,kw OR ("Therap*, Alternative"):ti,ab,kw OR ("electric-acupuncture"):ti,ab,kw OR ("electro-acupuncture"):ti,ab,kw OR ("warm acupuncture"):ti,ab,kw OR ("scalp acupuncture"):ti,ab,kw OR ("Integrat* Therap*"):ti,ab,kw 25210

#30 #28 OR #29 44593

#31 MeSH descriptor: [Randomized Controlled Trial] explode all trees 25733

#32 MeSH descriptor: [Randomized Controlled Trials as Topic] explode all trees 47399

#33 MeSH descriptor: [Controlled Clinical Trial] explode all trees 38478

#34 #31 OR #32 OR #33 85401

#35 (“Randomized Controlled Trial”):ti,ab,kw OR (“Randomized Controlled Trials as Topic”):ti,ab,kw OR (“Controlled Clinical Trial”):ti,ab,kw OR (“Randomized”):ti,ab,kw OR (“placebo”):ti,ab,kw OR (“randomly”):ti,ab,kw OR (“trial”):ti,ab,kw OR (“groups”):ti,ab,kw OR (“Clinical Trials”):ti,ab,kw OR (“Random”):ti,ab,kw OR (“RCT”):ti,ab,kw OR (“randomized controlled study”):ti,ab,kw OR (“randomized trial”):ti,ab,kw OR (“randomized study”):ti,ab,kw OR (“randomized placebo-controlled study”):ti,ab,kw OR (“randomized parallel-group study”):ti,ab,kw OR (“randomized placebo controlled”):ti,ab,kw OR (“randomized double-blin*”):ti,ab,kw 1506170

#36 #34 OR #35 1506200

#37 #17 AND #30 AND #36 33

n = 33

**Web of Science**

# Web of Science 检索策略 (v0.1)

# 数据库: Web of Science 核心合集

# 权限:

- WOS.SCI: 1970 to 2023

# 检索:

1: TS=("Constipation" OR "Dyschezia" OR "Colonic Inertia" OR "Astriction") 运行日期: Wed May 10 2023 12:01:32 GMT+0800 (中国标准时间) 检索结果: 27216

2: TS=("Stroke" OR "Strokes" OR "Cerebrovascular Accident" OR "Cerebrovascular Accidents" OR "CVA (Cerebrovascular Accident)" OR "CVAs (Cerebrovascular Accident)" OR "Cerebrovascular Apoplexy" OR "Apoplexy, Cerebrovascular" OR "Vascular Accident, Brain" OR "Brain Vascular Accident" OR "Brain Vascular Accidents" OR "Vascular Accidents, Brain" OR "Cerebrovascular Stroke" OR "Cerebrovascular Strokes" OR "Stroke, Cerebrovascular" OR "Strokes, Cerebrovascular" OR "Apoplexy" OR "Cerebral Stroke" OR "Cerebral Strokes" OR "Stroke, Cerebral" OR "Strokes, Cerebral" OR "Stroke, Acute" OR "Acute Stroke" OR "Acute Strokes" OR "Strokes, Acute" OR "Cerebrovascular Accident, Acute" OR "Acute Cerebrovascular Accident" OR "Acute Cerebrovascular Accidents" OR "Cerebrovascular Accidents, Acute" OR "Cerebral Infarction" OR "Cerebral Infarctions" OR "Infarctions, Cerebral" OR "Infarction, Cerebral" OR "Cerebral Infarct" OR "Cerebral Infarcts" OR "Infarct, Cerebral" OR "Infarcts, Cerebral" OR "Cerebral Infarction, Left Hemisphere" OR "Left Hemisphere, Cerebral Infarction" OR "Infarction, Cerebral, Left Hemisphere" OR "Cerebral, Left Hemisphere, Infarction" OR "Infarction, Left Hemisphere, Cerebral" OR "Left Hemisphere, Infarction, Cerebral" OR "Subcortical Infarction" OR "Infarction, Subcortical" OR "Infarctions, Subcortical" OR "Subcortical Infarctions" OR "Posterior Choroidal Artery Infarction" OR "Anterior Choroidal Artery Infarction" OR "Cerebral Infarction, Right Hemisphere" OR "Right Hemisphere, Cerebral Infarction" OR "Infarction, Right Hemisphere, Cerebral" OR "Right Hemisphere, Infarction, Cerebral" OR "Cerebral, Right Hemisphere, Infarction" OR "Infarction, Cerebral, Right Hemisphere" OR "Cerebral Hemorrhage" OR "Hemorrhage, Cerebrum" OR "Cerebrum Hemorrhage" OR "Cerebrum Hemorrhages" OR "Hemorrhages, Cerebrum" OR "Cerebral Parenchymal Hemorrhage" OR "Cerebral Parenchymal Hemorrhages" OR "Hemorrhage, Cerebral Parenchymal" OR "Hemorrhages, Cerebral Parenchymal" OR "Parenchymal Hemorrhage, Cerebral" OR "Parenchymal Hemorrhages, Cerebral" OR "Intracerebral Hemorrhage" OR "Hemorrhage, Intracerebral" OR "Hemorrhages, Intracerebral" OR "Intracerebral Hemorrhages" OR "Hemorrhage, Cerebral" OR "Cerebral Hemorrhages" OR "Hemorrhages, Cerebral" OR "Brain Hemorrhage, Cerebral" OR "Brain Hemorrhages, Cerebral" OR "Cerebral Brain Hemorrhage" OR "Cerebral Brain Hemorrhages" OR "Hemorrhage, Cerebral Brain" OR "Hemorrhages, Cerebral Brain" OR "Hemorrhagic Stroke" OR "Hemorrhagic Strokes" OR "Stroke, Hemorrhagic" OR "Subarachnoid Hemorrhagic Stroke" OR "Hemorrhagic Stroke, Subarachnoid" OR "Stroke, Subarachnoid Hemorrhagic" OR "Subarachnoid Hemorrhagic Strokes" OR "Intracerebral Hemorrhagic Stroke" OR "Hemorrhagic Stroke, Intracerebral" OR "Intracerebral Hemorrhagic Strokes" OR "Stroke, Intracerebral Hemorrhagic" OR "Intracerebral Hemorrhage Stroke" OR "Hemorrhage Stroke, Intracerebral" OR "Intracerebral Hemorrhage Strokes" OR "Stroke, Intracerebral Hemorrhage" OR "Ischemic Stroke" OR "Ischemic Strokes" OR "Stroke, Ischemic" OR "Ischaemic Stroke" OR "Ischaemic Strokes" OR "Stroke, Ischaemic" OR "Cryptogenic Ischemic Stroke" OR "Cryptogenic Ischemic Strokes" OR "Ischemic Stroke, Cryptogenic" OR "Stroke, Cryptogenic Ischemic" OR "Cryptogenic Stroke" OR "Cryptogenic Strokes" OR "Stroke, Cryptogenic" OR "Cryptogenic Embolism Stroke" OR "Cryptogenic Embolism Strokes" OR "Embolism Stroke, Cryptogenic" OR "Stroke, Cryptogenic Embolism" OR "Wake-up Stroke" OR "Stroke, Wake-up" OR "Wake up Stroke" OR "Wake-up Strokes" OR "Acute Ischemic Stroke" OR "Acute Ischemic Strokes" OR "Ischemic Stroke, Acute" OR "Stroke, Acute Ischemic" OR "Brain ischemia" OR "Brain Ischemias" OR "Ischemia, Brain" OR "Ischemic Encephalopathy" OR "Encephalopathy, Ischemic" OR "Ischemic Encephalopathies" OR "Cerebral Ischemia" OR "Cerebral Ischemias" OR "Ischemias, Cerebral" OR "Ischemia, Cerebral" OR "Brain Infarction" OR "Brain Infarctions" OR "Infarction, Brain" OR "Infarctions, Brain" OR "Brain Infarct" OR "Brain Infarcts" OR "Infarct, Brain" OR "Infarcts, Brain" OR "Brain Infarction, Posterior Circulation" OR "Infarction, Posterior Circulation, Brain" OR "Posterior Circulation Infarction, Brain" OR "Infarction, Brain, Posterior Circulation" OR "Posterior Circulation Brain Infarction" OR "Anterior Circulation Brain Infarction" OR "Infarction, Brain, Anterior Circulation" OR "Brain Infarction, Anterior Circulation" OR "Anterior Circulation Infarction, Brain" OR "Infarction, Anterior Circulation, Brain" OR "Venous Infarction, Brain" OR "Brain Venous Infarction" OR "Brain Venous Infarctions" OR "Infarction, Brain Venous" OR "Infarctions, Brain Venous" OR "Venous Infarctions, Brain" OR "Brain Infarction, Venous" OR "Brain Infarctions, Venous" OR "Infarction, Venous Brain" OR "Infarctions, Venous Brain" OR "Venous Brain Infarction" OR "Venous Brain Infarctions" OR "Anterior Cerebral Circulation Infarction" OR "Infarction, Anterior Cerebral Circulation" OR "Cerebrovascular Disorders" OR "Cerebrovascular Disorder" OR "Cerebrovascular Diseases" OR "Cerebrovascular Disease" OR "Disease, Cerebrovascular" OR "Diseases, Cerebrovascular" OR "Vascular Diseases, Intracranial" OR "Intracranial Vascular Disease" OR "Intracranial Vascular Diseases" OR "Vascular Disease, Intracranial" OR "Brain Vascular Disorders" OR "Brain Vascular Disorder" OR "Vascular Disorder, Brain" OR "Vascular Disorders, Brain" OR "Intracranial Vascular Disorders" OR "Intracranial Vascular Disorder" OR "Vascular Disorder, Intracranial" OR "Vascular Disorders, Intracranial" OR "Cerebrovascular Insufficiency" OR "Cerebrovascular Insufficiencies" OR "Insufficiencies, Cerebrovascular" OR "Insufficiency, Cerebrovascular" OR "Cerebrovascular Occlusion" OR "Cerebrovascular Occlusions" OR "Occlusion, Cerebrovascular" OR "Occlusions, Cerebrovascular" OR "CVA" OR "Intracranial Thromb*" OR "Ischemic Event " OR "Brain Thrombosis" OR "Cerebral Thrombus" OR "Ischemic Encephalopathies" OR "Ischemic Attack") 运行日期: Wed May 10 2023 12:02:16 GMT+0800 (中国标准时间) 检索结果: 474990

3: #1 AND #2 运行日期: Wed May 10 2023 12:02:50 GMT+0800 (中国标准时间) 检索结果: 226

4: TS=("Post stroke constipation" OR "Poststroke constipation") 运行日期: Wed May 10 2023 12:03:46 GMT+0800 (中国标准时间) 检索结果: 12

5: #3 OR #4 运行日期: Wed May 10 2023 12:03:56 GMT+0800 (中国标准时间) 检索结果: 227

6: TS=("Acupuncture" OR "Acupuncture Therapy" OR "Acupuncture Treatment*" OR "Treatment, Acupuncture" OR "Therapy, Acupuncture" OR "Pharmacoacupuncture Treatment" OR "Treatment, Pharmacoacupuncture" OR "Pharmacoacupuncture Therapy" OR "Therapy, Pharmacoacupuncture" OR "Acupotom*" OR "Acupuncture*, Ear" OR "Ear Acupuncture*" OR "Auricular Acupuncture*" OR "Acupuncture*, Auricular" OR "Acupuncture Point*" OR "Point*, Acupuncture" OR "Acupoint*" OR "Auriculotherap*" OR "electroacupuncture" OR "Dry Needling" OR "Needling, Dry" OR "moxibustion" OR "Moxabustion" OR "meridians" OR "Ching Lo" OR "Jing Luo" OR "Luo, Jing" OR "Jingluo" OR "Complementary Therap*" OR "Therap*, Complementary" OR "Complementary Medicine" OR "Medicine, Complementary" OR "Alternative Medicine" OR "Medicine, Alternative" OR "Alternative Therap*" OR "Therap*, Alternative" OR "electric-acupuncture" OR "electro-acupuncture" OR "warm acupuncture" OR "scalp acupuncture" OR "Integrat* Therap*") 运行日期: Wed May 10 2023 12:06:53 GMT+0800 (中国标准时间) 检索结果: 63923

7: TS=(“Randomized Controlled Trial” OR “randomized controlled trial (topic) ” OR “controlled clinical trial” OR “Randomized” OR “placebo” OR “randomly” OR “trial” OR “groups” OR “Clinical Trials” OR “Random” OR “RCT” OR “randomized controlled study” OR “randomized trial” OR “randomized study” OR “randomized placebo-controlled study” OR “randomized parallel-group study” OR “randomized placebo controlled” OR “randomized double-blind”) 运行日期: Wed May 10 2023 12:07:29 GMT+0800 (中国标准时间) 检索结果: 4865892

8: #5 AND #6 AND #7 运行日期: Wed May 10 2023 12:07:57 GMT+0800 (中国标准时间) 检索结果: 9

n = 9

**Chinese Clinical Trial Register**

n = 1

**ClinicalTrials.gov**

n = 3

## Supplementary File 2. The PRISMA checklist of this meta-analysis.

| **Section and Topic** | **Item #** | **Checklist item** | **Location where item is reported** |
| --- | --- | --- | --- |
| **TITLE** | | |  |
| Title | 1 | Identify the report as a systematic review. | P1 |
| **ABSTRACT** | | |  |
| Abstract | 2 | See the PRISMA 2020 for Abstracts checklist. | P1-P2 |
| **INTRODUCTION** | | |  |
| Rationale | 3 | Describe the rationale for the review in the context of existing knowledge. | P2 |
| Objectives | 4 | Provide an explicit statement of the objective(s) or question(s) the review addresses. | P2 |
| **METHODS** | | |  |
| Eligibility criteria | 5 | Specify the inclusion and exclusion criteria for the review and how studies were grouped for the syntheses. | P3-P4 |
| Information sources | 6 | Specify all databases, registers, websites, organisations, reference lists and other sources searched or consulted to identify studies. Specify the date when each source was last searched or consulted. | P3 |
| Search strategy | 7 | Present the full search strategies for all databases, registers and websites, including any filters and limits used. | P3 and Supplementary File 1 |
| Selection process | 8 | Specify the methods used to decide whether a study met the inclusion criteria of the review, including how many reviewers screened each record and each report retrieved, whether they worked independently, and if applicable, details of automation tools used in the process. | P4 |
| Data collection process | 9 | Specify the methods used to collect data from reports, including how many reviewers collected data from each report, whether they worked independently, any processes for obtaining or confirming data from study investigators, and if applicable, details of automation tools used in the process. | P4 |
| Data items | 10a | List and define all outcomes for which data were sought. Specify whether all results that were compatible with each outcome domain in each study were sought (e.g. for all measures, time points, analyses), and if not, the methods used to decide which results to collect. | P4 |
|  | 10b | List and define all other variables for which data were sought (e.g. participant and intervention characteristics, funding sources). Describe any assumptions made about any missing or unclear information. | P4 |
| Study risk of bias assessment | 11 | Specify the methods used to assess risk of bias in the included studies, including details of the tool(s) used, how many reviewers assessed each study and whether they worked independently, and if applicable, details of automation tools used in the process. | P4 |
| Effect measures | 12 | Specify for each outcome the effect measure(s) (e.g. risk ratio, mean difference) used in the synthesis or presentation of results. | P4-P5 |
| Synthesis methods | 13a | Describe the processes used to decide which studies were eligible for each synthesis (e.g. tabulating the study intervention characteristics and comparing against the planned groups for each synthesis (item #5)). | P4-P5 |
|  | 13b | Describe any methods required to prepare the data for presentation or synthesis, such as handling of missing summary statistics, or data conversions. | P4-P5 |
|  | 13c | Describe any methods used to tabulate or visually display results of individual studies and syntheses. | P4-P5 |
|  | 13d | Describe any methods used to synthesize results and provide a rationale for the choice(s). If meta-analysis was performed, describe the model(s), method(s) to identify the presence and extent of statistical heterogeneity, and software package(s) used. | P4-P5 |
|  | 13e | Describe any methods used to explore possible causes of heterogeneity among study results (e.g. subgroup analysis, meta-regression). | P4-P5 |
|  | 13f | Describe any sensitivity analyses conducted to assess robustness of the synthesized results. | P4-P5 |
| Reporting bias assessment | 14 | Describe any methods used to assess risk of bias due to missing results in a synthesis (arising from reporting biases). | P4-P5 |
| Certainty assessment | 15 | Describe any methods used to assess certainty (or confidence) in the body of evidence for an outcome. | P5 |
| **RESULTS** | | |  |
| Study selection | 16a | Describe the results of the search and selection process, from the number of records identified in the search to the number of studies included in the review, ideally using a flow diagram. | P5 and Figure 1 |
|  | 16b | Cite studies that might appear to meet the inclusion criteria, but which were excluded, and explain why they were excluded. | Supplementary Table 1 |
| Study characteristics | 17 | Cite each included study and present its characteristics. | P5 and Table1 |
| Risk of bias in studies | 18 | Present assessments of risk of bias for each included study. | P8-9 and Figure 3 |
| Results of individual studies | 19 | For all outcomes, present, for each study: (a) summary statistics for each group (where appropriate) and (b) an effect estimate and its precision (e.g. confidence/credible interval), ideally using structured tables or plots. | P9-P11 and Figure 4-13 |
| Results of syntheses | 20a | For each synthesis, briefly summarise the characteristics and risk of bias among contributing studies. | P9-P11 and Figure 4-13 |
|  | 20b | Present results of all statistical syntheses conducted. If meta-analysis was done, present for each the summary estimate and its precision (e.g. confidence/credible interval) and measures of statistical heterogeneity. If comparing groups, describe the direction of the effect. | P9-P11 and Figure 4-13 |
|  | 20c | Present results of all investigations of possible causes of heterogeneity among study results. | P9-P11 and Figure 4-13 |
|  | 20d | Present results of all sensitivity analyses conducted to assess the robustness of the synthesized results. | P9-P11 and Supplementary Figure 1 |
| Reporting biases | 21 | Present assessments of risk of bias due to missing results (arising from reporting biases) for each synthesis assessed. | P11, Figure 14, and Supplementary Table 4 |
| Certainty of evidence | 22 | Present assessments of certainty (or confidence) in the body of evidence for each outcome assessed. | P11 and Table 2 |
| **DISCUSSION** | | |  |
| Discussion | 23a | Provide a general interpretation of the results in the context of other evidence. | P14-15 |
|  | 23b | Discuss any limitations of the evidence included in the review. | P15 |
|  | 23c | Discuss any limitations of the review processes used. | P15 |
|  | 23d | Discuss implications of the results for practice, policy, and future research. | P15 |
| **OTHER INFORMATION** | | |  |
| Registration and protocol | 24a | Provide registration information for the review, including register name and registration number, or state that the review was not registered. | P2 |
|  | 24b | Indicate where the review protocol can be accessed, or state that a protocol was not prepared. | P2 |
|  | 24c | Describe and explain any amendments to information provided at registration or in the protocol. | P2 |
| Support | 25 | Describe sources of financial or non-financial support for the review, and the role of the funders or sponsors in the review. | P16 |
| Competing interests | 26 | Declare any competing interests of review authors. | P16 |
| Availability of data, code and other materials | 27 | Report which of the following are publicly available and where they can be found: template data collection forms; data extracted from included studies; data used for all analyses; analytic code; any other materials used in the review. | P16 |

*From:*  Page MJ, McKenzie JE, Bossuyt PM, Boutron I, Hoffmann TC, Mulrow CD, et al. The PRISMA 2020 statement: an updated guideline for reporting systematic reviews. BMJ 2021;372:n71. doi: 10.1136/bmj.n71

For more information, visit: <http://www.prisma-statement.org/>
